# Completeness of open access FluNet influenza surveillance data for Pan-America in 2005–2019

**DOI:** 10.1038/s41598-020-80842-9

**Published:** 2021-01-12

**Authors:** Ryan B. Simpson, Jordyn Gottlieb, Bingjie Zhou, Meghan A. Hartwick, Elena N. Naumova

**Affiliations:** Tufts University Friedman School of Nutrition Science and Policy, Boston, USA

**Keywords:** Influenza virus, Diseases, Risk factors, Disease prevention, Health policy, Epidemiology

## Abstract

For several decades, the World Health Organization has collected, maintained, and distributed invaluable country-specific disease surveillance data that allow experts to develop new analytical tools for disease tracking and forecasting. To capture the extent of available data within these sources, we proposed a completeness metric based on the effective time series length. Using FluNet records for 29 Pan-American countries from 2005 to 2019, we explored whether completeness was associated with health expenditure indicators adjusting for surveillance system heterogeneity. We observed steady improvements in completeness by 4.2–6.3% annually, especially after the A(H1N1)-2009 pandemic, when 24 countries reached > 95% completeness. Doubling in decadal health expenditure per capita was associated with ~ 7% increase in overall completeness. The proposed metric could navigate experts in assessing open access data quality and quantity for conducting credible statistical analyses, estimating disease trends, and developing outbreak forecasting systems.

## Introduction

Global and national surveillance systems serve two critical functions: monitoring disease trend trajectories to inform health policies and providing early outbreak warnings that require local, regional, or global responses^1^. Extensive time, personnel, and monetary resources are required to collect, process, and maintain time referenced and geographically tagged surveillance data. These data enable a rapid evidence-based response to protect human health, distribute supplies, and mitigate disease outbreaks. Effective surveillance data must be credible to produce reliable alerts, complex to incorporate a variety of data streams, and historically rich to track disease trajectories for early warning detection^2^. Poor-quality surveillance data can lead to policy interventions that are based on inaccurately interpreted patterns resulting in diminished quality of life, less productive societies, and slower global responses^3,4^. High quality data available to a broad range of experts are critical to reliably predict disease trends^2^.

The World Health Organization (WHO) has played an instrumental role in regulating the generation of international surveillance data for over 70 years. The WHO has established high standards by using a comprehensive set of monitoring and evaluation (M&E) metrics to routinely collect worldwide records^5,6^. These metrics track the production of surveillance data and provide critical information for effective analysis and interpretation of the data. Some of these metrics like *sensitivity*, *specificity*, and *positive predicted value* measure the accuracy and reliability of testing protocols and mechanisms. Other metrics, like *timeliness* and *representativeness*, provide information on the frequency and comprehensiveness of data incorporated within surveillance systems^6^. Together, these M&E metrics help data users understand the reliability of patterns that are captured, detected, and demonstrated based on the collected data.

The WHO established the Global Influenza Surveillance Network (GISN) in 1952 to raise awareness of the economic impact and public health consequences of influenza^7–9^. In 1997, the standardization of polymerase chain reaction (PCR) technology enabled rapid case identification of influenza infections and improved the ability to definitively diagnose influenza infection. These scientific breakthroughs made global virological influenza surveillance possible and led to a strengthening of the GISN through the creation of FluNet: a system of over 122 national influenza centers (NICs) and 6 international centers interconnected by Internet servers that consistently record population-level influenza in over 170 countries^9–12^. FluNet data is publicly available to encourage wide dissemination and analysis of influenza trends, burdens, and patterns^12^. This public platform supports the broader mission of WHO influenza surveillance: to monitor, plan for, and alert the world on novel influenza epidemiology for seasonal, pandemic, and zoonotic influenza^12^.

As the velocity and volume of collected data increased from 1998–2010, so did opportunities to utilize multiple data streams and disseminate surveillance records more broadly. This gave rise to web-based platforms like FluID that actively collect, deposit, and report influenza health records using various influenza case definitions and surveillance strategies^13^. Information reported by these platforms includes numerous influenza-related case definitions, testing techniques, surveillance strategies, reporting timeliness, and population coverage. Once registered, any certified health center, not just a NIC, is able to participate in this data curation. The information collected by platforms like FluID complement FluNet to improve the accuracy and coverage of estimating true influenza incidence^9^. The merging of multiple data streams has been shown to improve rates of influenza testing and diagnosis, which greatly influence the reporting completeness of surveillance data^3,4,14^.

National healthcare infrastructure and public health resources are likely to drive the reliability, completeness, and accuracy of reported data^5^. Thus, FluNet relies on the case identification and collection capacity of each participating country. While several studies examined the association between country wealth and the burden of influenza^15–17^, little is known whether country income or health expenditure indicators along with the national surveillance system attributes influence data availability. A broad network of available data streams might facilitate data collection and reporting to FluNet, but does not guarantee data completeness. Furthermore, existing WHO M&E metrics that target surveillance system quality are not embedded into metadata of publicly available records. This questions whether and how external users assess the quality and completeness of available data^18,19^. Yet, the completeness of publicly disseminated surveillance data influences modeled disease trends, seasonal features, outbreak signatures, and forecasts^20–23^.

In this communication, we proposed a metric of *completeness* based on the effective time series length (ETSL) to capture the extent of the available time series data within FluNet records. We illustrated the utility of this metric for 29 Pan-American countries across 14 influenza variables (6 testing outcomes and 8 strain subtypes) from 2005 to 2019. We calculated this metric for each country using annual (52–53 weeks), full study (782 weeks), and select interval (470–782 weeks) time period lengths. We ranked countries based on completeness estimates and determined trends across countries. We adjusted completeness estimates for specific strain subtypes (e.g. A(H1N1)pdm09) to isolate only time periods when reporting is meaningful. We applied a mixed effects regression model to evaluate whether national economic indicators could explain the degree of completeness for each influenza variable. Our proposed completeness metric helps external data users understand the amount of data available for analyses and the potential of data to accurately estimate disease trends, detect temporal changes, and develop spot checks in data quality. This metric can also assist data users to recognize data limitations, understand the heterogeneity of primary data sources, and develop strategies for conducting credible statistical analyses using publicly disseminated surveillance data. The presented material is especially important in light of publicly reported time series data for the ongoing coronavirus disease 2019 (COVID-19) pandemic.

## Data and methods

### FluNet weekly records

We abstracted FluNet weekly records on 24–27 April 2020 for 29 Pan American countries from Week 1 (03 January) 2005 through Week 52 (29 December) 2019. Due to the absence of a public application programming interface (API) and the challenges of the website’s dynamic AJAX interface^24^, we acquired public data with a custom scraper built using RSelenium^25^. We downloaded each country’s records individually and used a scripted pipeline to standardize and merge country-specific datasets. Codes are available in the Supplementary Materials Appendix.

FluNet reports time series data of influenza confirmed cases representing a complex array of data streams. For each country download, we extracted time series data for 6 available testing outcomes: specimens collected, specimens processed (tests), total positives, total negatives, influenza A positives, and influenza B positives. We also extracted time series data for 8 influenza subtypes: A(H1), A(H1N1)pdm09, A(H3), A(H5), A(Unsubtyped), B(Yamataga), B(Victoria), and B(Undetermined). The overall data compilation included 14 variables for 29 countries covering 782 weeks.

### Surveillance systems attributes

We compiled FluNet case definitions, surveillance strategies, reporting quota and timeliness, NICs, and reporting facilities using several WHO reports^6,26–28^ to assess surveillance attributes associated with completeness (Table 1). Influenza case definitions are not fully standardized: they have subtle but important differences in their evaluation setting and diagnostic criteria. Case definitions for each country include severe acute respiratory illness (SARI), influenza-like-illness (ILI), pneumonia, influenza cases (Influenza), acute respiratory infection (ARI), and deaths (Mortality) (Supplementary Table S1). Mortality was defined as deaths from influenza unless otherwise specified. Six FluNet countries (Chile, Cuba, Dominica, Dominican Republic, El Salvador, and Honduras) have fully adopted the WHO case definitions. We marked countries utilizing non-WHO definitions.

**Table 1:** Case definitions, adherence to WHO case definition, surveillance strategies, reporting quotas, reporting timeframes, number of NICs, and reporting facilities for 29 Pan-American FluNet-reporting countries based on WHO and PAHO reports from 2017^10–13^.

Country (abbreviation)	Case definition	WHO definition^a^	Surveillance strategy^a^	Reporting quota^b^	Reporting timeframe^c^	NICs	Reporting facilities
Argentina (ARG)	SARI	Yes	Sentinel	All cases	Weekly	3	44
		Yes	Universal	All cases	Weekly		
	Pneumonia	No	NR	NR	NR		
	Mortality	Yes	Universal	All cases	Weekly		
	ILI	Yes	Universal	0	NR		
		Yes	Sentinel	Unknown	Weekly		
Barbados (BRB)	SARI	No	Sentinel	All cases	Daily	0	3
	Influenza	Yes	Sentinel	All cases	NR		
	ARI	No	Sentinel	6	NR		
Belize (BLZ)	SARI	Yes	Sentinel	All cases	NR	0	10
	Influenza	NR	National	All cases	Weekly		
	ILI	Yes	National	0	NR		
Bolivia (BOL)	SARI	Yes	Sentinel	All cases	Weekly	0	11
	Pneumonia	No	National	0	NR		
	Influenza	NR	National	All cases	Weekly		
Brazil (BRA)	SARI	No	Sentinel	All cases	Daily	3	258
	Mortality	Yes	NR	NR	NR		
	Influenza	Yes	National	All cases	Daily		
	ILI	No	Sentinel	All cases	Daily		
Canada (CAN)	SARI	Yes	Sentinel	Varies	Varies	11	147
	Pneumonia	No	National	Varies	Varies		
	Influenza	Yes	National	All cases	Varies		
	ILI	Yes	Sentinel	Varies	Weekly		
	ARI	No	National	Varies	Varies		
Chile (CHL)	SARI	Yes	Sentinel	All cases	Weekly	1	79
	Influenza	Yes	National	All cases	Daily		
	ILI	Yes	Sentinel	10	Weekly		
Colombia (COL)	SARI	Yes	Sentinel	All cases	Weekly	1	32
	Pneumonia	No	National	0	NR		
	Mortality	No	National	All cases	Weekly		
	Influenza	NR	NR	NR	NR		
	ILI	No	Sentinel	All cases	Weekly		
	ARI	No	National	0	NR		
Costa Rica (CRI)	SARI	Yes	Sentinel	5	Weekly	1	21
	Pneumonia	No	Universal	Unknown	NR		
	Mortality	Yes	Universal	Unknown	NR		
	Influenza	NR	Sentinel	5	Daily		
	ILI	Yes	Universal	NR	NR		
	ARI	No	National	Unknown	NR		
Cuba (CUB)	SARI	Yes	National	All cases	Daily	1	154
	Pneumonia	Yes	National	All cases	Daily		
	Mortality	Yes	National	NR	NR		
	ILI	Yes	National	Unknown	Daily		
	ARI	Yes	National	Unknown	Daily		
Dominica (DMA)	SARI	Yes	Sentinel	All cases	Weekly	0	1
	Mortality	Yes	National	0	NR		
	ARI	Yes	National	6	Weekly		
Dominican Republic (DOM)	SARI	Yes	Sentinel	5	Weekly	1	12
	Influenza	Yes	Sentinel	All cases	Weekly		
	ILI	Yes	Sentinel	5	Weekly		
Ecuador (ECU)	SARI	Yes	Sentinel	All cases	Daily	1	18
	Pneumonia	No	Universal	0	NR		
	Influenza	NR	National	NR	NR		
El Salvador (SLV)	SARI	Yes	Sentinel	5	Weekly	1	14
	ILI	Yes	Sentinel	3	Weekly		
Guatemala (GTM)	SARI	Yes	Sentinel	6	Weekly	1	9
	Pneumonia	No	Universal	0	NR		
	Influenza	NR	National	NR	NR		
	ILI	Yes	Sentinel	6	Weekly		
	ARI	No	National	0	NR		
Haiti (HTI)	SARI	Yes	Sentinel	10	NR	0	15
	ARI	NR	National	0	NR		
Honduras (HND)	SARI	Yes	Sentinel	All cases	Weekly	1	10
	Pneumonia	Yes	National	NR	NR		
	Mortality	Yes	Sentinel	NR	NR		
		Yes	National	NR	NR		
	Influenza	Yes	Sentinel	All cases	Weekly		
	ILI	Yes	Sentinel	7	Weekly		
	ARI	Yes	National	NR	NR		
		Yes	Sentinel	NR	NR		
Jamaica (JAM)	SARI	Yes	Sentinel	All cases	Weekly	1	6
	Influenza	Yes	National	All cases	Daily		
	ARI	NR	Sentinel	All cases	Daily		
Mexico (MEX)	Influenza	Yes	Sentinel	All cases	Weekly	1	995
	ILI	No	Sentinel	Varies	Weekly		
	ARI	No	Sentinel	All cases	Weekly		
Nicaragua (NIC)	SARI	Yes	Sentinel	All cases	Weekly	1	17
	Pneumonia	No	National	0	NR		
	Mortality	Yes	National	0	NR		
	Influenza	NR	Sentinel	All cases	Weekly		
	ILI	Yes	Sentinel	3	Weekly		
	ARI	No	National	0	NR		
Panamá (PAN)	SARI	Yes	Sentinel	Varies	Weekly	1	24
	Pneumonia	NR	National	NR	NR		
	Influenza	Yes	National	Unknown	Daily		
		Yes	Sentinel	Unknown	Daily		
	ILI	Yes	Sentinel	Varies	Weekly		
	ARI	No	National	NR	NR		
Paraguay (PRY)	SARI	No	Sentinel	All cases	Weekly	1	17
	Pneumonia	No	Universal	0	NR		
	ILI	No	Sentinel	5	Weekly		
	ARI	No	National	0	NR		
Peru (PER)	SARI	Yes	Sentinel	All cases	Daily	1	46
	Pneumonia	NR	Universal	0	NR		
	Mortality	Yes	National	0	NR		
	ILI	Yes	Sentinel	Unknown	Weekly		
St. Lucia (LCA)	SARI	Yes	Sentinel	All cases	Weekly	0	41
	Influenza	Yes	National	6	NR		
	ILI	Yes	Sentinel	6	NR		
	ARI	No	National	All cases	NR		
St. Vincent & the Grenadines (VCT)	SARI	Yes	Sentinel	All cases	Daily	0	1
	ILI	NR	National	NR	NR		
Suriname (SUR)	SARI	Yes	Sentinel	All cases	Daily	0	7
	Pneumonia	NR	National	0	NR		
	Mortality	Yes	National	0	NR		
	ILI	Yes	Sentinel	10	Weekly		
	ARI	No	Sentinel	0	NR		
Uruguay (URY)	SARI	Yes	Sentinel	All cases	Weekly	1	44
	Influenza	NR	Sentinel	All cases	Weekly		
United States (USA)	SARI	No	Sentinel	All cases	NR	1	3100
	Influenza	Yes	National	All cases	Weekly		
	ILI	NR	Sentinel	Unknown	Weekly		
Venezuela (VEN)	Influenza	NR	National	All cases	Weekly	1	NR

^a^NR – country case definitions or surveillance systems that were not reported or unavailable within WHO and PAHO reports.

^b^Quotas listed as *All Cases* report all surveillance cases to FluNet. Numbers ranging from 0 to 10 list the number of cases per reporting timeframe reported to FluNet. Quotas listed as *Varies* indicates that quotas are a percentage of the total cases reported or that quotas fluctuate depending on case severity. For quotas listed as *Unknown*, a quota exists but values are not provided in WHO and PAHO reports. Quotas listed as *NR* had neither the existence nor the quantity of a quota reported in WHO and PAHO reports.

^c^Reporting timeframe indicates the delay between collecting and processing test shipments. Timeframes reported as *Varies* suggests that this process changes over time. Timeframes that are listed as NR were unavailable within WHO and PAHO reports.

Surveillance strategies include national, sentinel, and universal, which offer different population coverage for each influenza case definition (Supplementary Table S2). NICs are nationally recognized institutions approved by the WHO and responsible for reporting influenza surveillance records to FluNet. Influenza reporting facilities include SARI hospitals, ILI centers, PCR testing facilities, and influenza (IF) testing laboratories, and their number vary by country. Each facility processes tests at different volumes and speeds, resulting in differing reporting quotas and timeframes. Reporting quotas describe the fraction of cases that are reported to FluNet from in-country surveillance systems. They include all cases or a specific number of cases based on each country’s health objectives. Reporting timeframe is the difference between when disease cultures are laboratory confirmed and when they are reported by surveillance facilities to a national or global database. Though FluNet publishes weekly records, reporting timeframes range from daily to monthly across countries. We found no resources that compile these surveillance system characteristics to allow for clear side-by-side comparison across continental countries.

### Economic and health expenditure indicators

We extracted three economic indicators for each country reported by the World Bank’s *World Development Indicators* publicly available database. Indicators included Gross National Income (GNI) per capita (GNIPC), domestic general government health expenditure per capita (DHEPC), and out-of-pocket health expenditure as a percentage of current health expenditure (OOPHE%)^29^. GNIPC was reported in purchasing power parity (PPP) constant 2011 international US dollars (USD). DHEPC was reported in current international USD. OOPHE% is reported as the percentage of current government expenditure (Supplementary Table S3). GNIPC estimates were available for all countries from 2005 to 2018 except Cuba and Venezuela (data available for 2005–2016 and 2005–2014, respectively). DHEPC and OOPHE% records were available from 2005 to 2016 for all countries except Venezuela (data available for 2005–2015).

### Completeness metric

We measured *completeness* based on an effective time series length (ETSL), or the extent of time series data that can be used in data analysis. Time series length greatly influenced how and what the completeness metric described. We used an annual ETSL to calculate completeness from Week 1 to Week 52/53 to compare completeness between outcomes and across countries. We used these values to calculate the overall completeness, or the mean across all years from 2005 to 2019. We also calculated the average completeness using the full time series ETSL for the entire 782-week study period. Finally, we calculated the corrected average values and corrected overall completeness by including only years when selected outcomes or subtypes were reported. This prevented the deflating of the completeness metric by including all study years irrespective of whether a country’s surveillance records were present or not.

We calculated the annual completeness, *C*_*i,j,k*_, as a fraction of the time series length for which reliable data are available to the overall length of the considered time series, or the number of full weeks between the start and end of the time period, multiplied by 100:

$$C_{{i,j,k}} = ~\left( {{\raise0.7ex\hbox{${n_{{i,j,k}} }$} \!\mathord{\left/ {\vphantom {{n_{{i,j,k}} } {L_{1} }}}\right.\kern-\nulldelimiterspace} \!\lower0.7ex\hbox{${L_{1} }$}}} \right)*100\%$$

where *C*_*i,j,k*_ is completeness for *i*-outcome (*i* = 1–14), *j*-country (*j* = 1–29), *k*-year (*k* = 1–15); *n*_*i,j,k*_—the number of time units (weeks) in the time series when records are available (e.g. weeks with reported counts ≥ 0) for *i*-outcome, *j*-country, *k*-year; *L*_1_—the number of full weeks (52 or 53) for *k*-year (Table 2). We calculated the annual completeness by using the total length in weeks for each year.

**Table 2:** Number of full weeks included for calculating the completeness metric using annual, full study, and corrected effective time series lengths.

Timeframe	Outcomes	ETSL reporting dates	Number of full weeks, L
Annual	All 14 outcomes	1 Jan–31 Dec	L_1_ = 52 or 53 52: 2005–2008, 2010–2014, 2016–2019 53: 2009, 2015
Average full study period	All 14 outcomes	03 Jan 2005–29 Dec 2019	L_2_ = 782
Average corrected	A(H1N1)pdm09 and Specimens A(H5) B(Yamataga) and B(Victoria)	31 Dec 2007–29 Dec 2019 31 Dec 2007–01 Jan 2017 01 Jan 2007–29 Dec 2019	L_3_ = 626 L_3_ = 470 L_3_ = 678

We calculated the completeness for each outcome and country for the full time series using the total 782-week length covering the study period from Week 1 (03 January) 2005 to Week 52 (29 December) 2019, as:

$${C}_{i,j}= \left(n_{i,j}/{L}_{i,2}\right)*100\%$$

where *C*_*i,j*_ is completeness for *i*-outcome (*i* = 1–14), *j*-country (*j* = 1–29); *n*_*i,j*_—the number of time units (weeks) in the time series when records are available (e.g. weeks with reported counts ≥ 0) for *i*-outcome, *j*-country; *L*_*i*_*,*_2_—the number of full weeks (782) or the full study period. To draw comparisons across countries, we calculated the overall completeness as the average completeness across all 14 outcomes for each country.

To more accurately reflect completeness for specific influenza outcomes, we corrected the average estimates to only include the time period when reporting is meaningful. For example, Jamaica, Paraguay, and Mexico were the first Pan American countries to report the new influenza subtype A(H1N1)pdm09 in 2008. All 29 countries have continued reporting this subtype as of 2019. Thus, we estimated the average estimates for A(H1N1)pdm09 to account for the start of pandemic strain reporting in 2008 for all countries. Specimens were also first reported in 2008 by Paraguay with Canada and the United States continuing reporting through 2019; completeness for specimens was similarly estimated for a 626-week time period from Week 1 2008 to Week 52 2019. For A(H5), we estimated average completeness from Week 1 2008 to Week 52 2016 (470 weeks). For B(Yamataga) and B(Victoria), we estimated average completeness for a 678-week time period from Week 1 2007 to Week 52 2019. All analyses of average estimates were performed using values of *L*_3_ as shown in Table 2.

### Completeness analysis

We examined trends in annual completeness for all 14 influenza variables and produced heatmaps illustrating the country ranking with respect to completeness. To further examine trends and associations with national economic indicators (GNIPC, DHEPC, and OOPHE%), we selected annual completeness estimates for tests, positives, A(H1N1)pdm09, and overall. We applied loess smoothers with a span of 0.5 to illustrate trends across all years and countries. We transformed the GNIPC and DHEPC values using the natural logarithm function to minimize the effect of skewed distributions in regression models. For each influenza variable, we estimated the change in annual completeness associated with time and national economic indicators using a mixed effects regression model (Model 1):

$${C}_{i,j,k}= {\beta }_{0}+ {\beta }_{1}*{Year}_{k}+ {\beta }_{2}*{E}_{j,k}+ {\alpha }_{j}*{Country}_{j}+ {\varepsilon }_{jk}$$

where *C*_*i,j,k*_ is completeness for *i*-outcome (*i* = 1–4), *j*-country (*j* = 1–29), *k*-year; *E*_*j,k*_ – one of three national indicators for *j*-country and *k*-year; *β*_1_—fixed effects for the annual trend, α_*j*_ – random effects for individual countries. The length of the time series used in each regression varied according to the length of available records for the economic or health expenditure indicator in each country.

We expanded the model to adjust for surveillance systems’ attributes (Model 2):

$${C}_{i,j,k}= {\beta }_{0}+ {\beta }_{1}*{Year}_{k}+ {\beta }_{2}*{E}_{j,k}+{\beta }_{m}*{S}_{j,m}+ {\alpha }_{j}*{Country}_{j }+ {\varepsilon }_{jk}$$

where *S*_*j,m*_—matrix of the national surveillance system attributes as defined in Table 1. Attributes included in the analysis were: case definition type, including ARI, ILI, Influenza, Pneumonia, Mortality, and SARI as the reference category; adherence to WHO definition as a binary variable; surveillance strategy, including Sentinel, National, and Universal as the reference category; reporting quota, including categories for reported quota, varying or unknown quota, and reporting all cases as the reference category; reporting timeframe, as weekly, not reported (NR), and daily reporting as the reference; number of NICs, and the natural log of reporting facilities number (we applied the transformation given the skewed distribution of reporting facilities as fewer countries have many facilities).

To estimate the effect size (ES) from regression model results, we calculated the decadal change between 2005 and 2015 for each economic and health expenditure indicator. For Venezuela, we estimated decadal changes in GNIPC from 2005 through 2014 due to limited data availability. Using the coefficients from Models 1 and 2, we estimated ES and its 95% confidence intervals (CI) associated with time and health expenditure indicators in the completeness of tests, positives, A(H1N1)pdm09, and overall completeness. For GNIPC and DHEPC, the effect size was associated with a doubling in these predictors; the 95% confidence interval was estimated as: $ES=ln\left(2\right)*\left({\beta }_{2}+1.96se\left({\beta }_{2}\right)\right)$. For OOPHE%, ES was associated with a 10% increase in expenditures; 95%CI was estimated as: $ES=10*\left({\beta }_{2}+1.96se\left({\beta }_{2}\right)\right)$.

We used Akaike Information Criterion (AIC) to assess model performance. Data management, statistical analyses, and maps were conducted using Stata/SE 15.1 and R versions 1.1.419 and 4.0.0.

## Results

### Annual completeness, trends, and spot-checking

**Figure 1:**
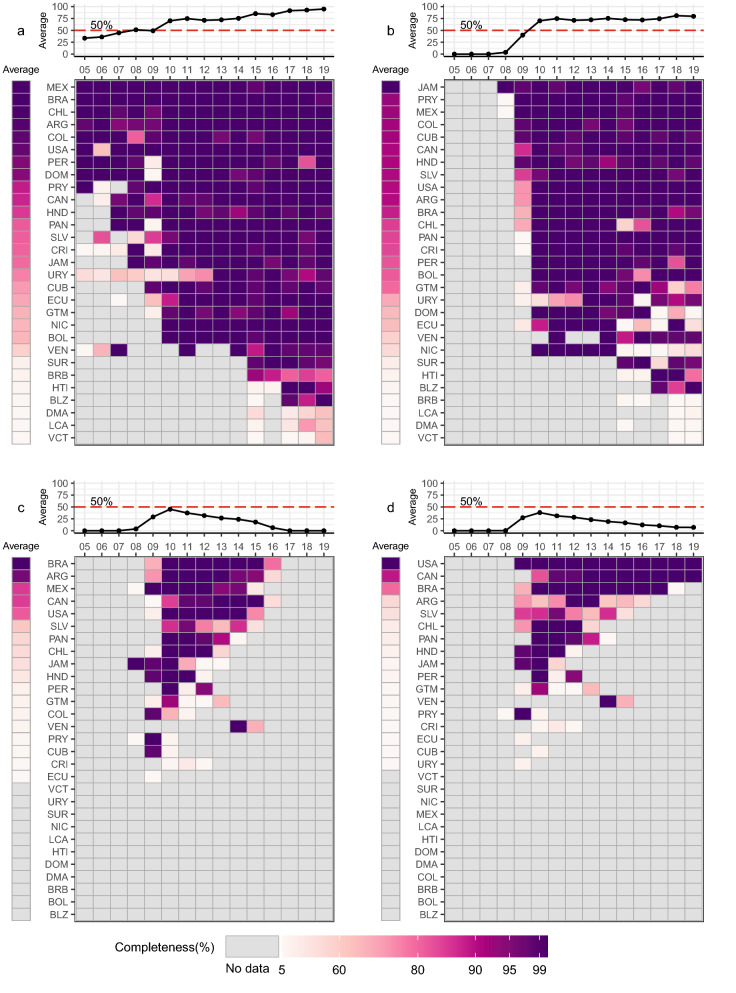
Multi-panel plots of annual completeness of (**a**) positives, (**b**) A(H1N1)pdm09, (**c**) A(H5), and (**d**) specimens for 29 Pan American countries from 2005 through 2019. The top panel provides a line plot of the average completeness across all countries with a dashed red line indicating 50% completeness. The bottom panel provides a heatmap of completeness with grey color indicating no data, white color indicating near 0% completeness, and purple color indicating near 100% completeness. Countries are listed in descending order by corrected average completeness using three letter country abbreviations.

**Figure 2:**
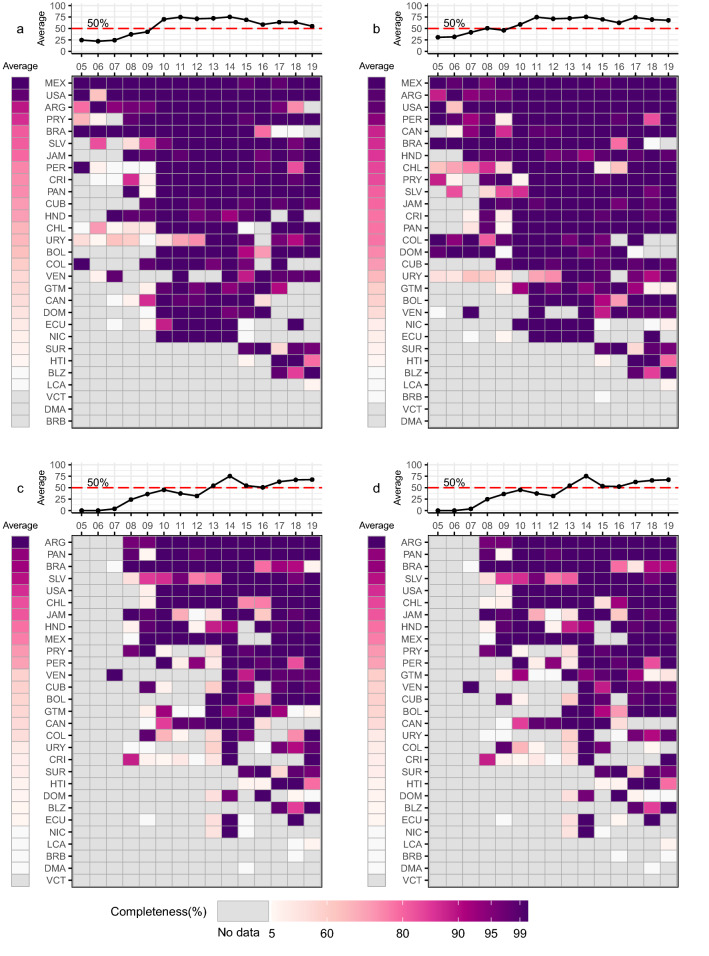
Multi-panel plots of annual completeness of (**a**) A(H1), (**b**) A(Unsubtyped), (**c**) B(Yamataga), and (**d**) B(Victoria) for 29 Pan American countries from 2005 through 2019. The top panel provides a line plot of the average completeness across all countries with a dashed red line indicating 50% completeness. The bottom panel provides a heatmap of completeness with grey color indicating no data, white color indicating near 0% completeness, and purple color indicating near 100% completeness. Countries are listed in descending order by corrected average completeness using three letter country abbreviations.

The annual completeness values for each influenza outcome in each country and year of the 15-year study period are compiled in Supplementary Table S4. Figures 1 and 2 show the completeness values for influenza outcomes along with the trend line for average completeness across all 29 countries presented as a heatmap and a line-plot, respectively. In each country, completeness for tests, positives, influenza A positives, and influenza B positives are almost identical (completeness for positives shown in Fig. 1a). In 2005, these 4 outcomes are nearly 100% complete for nine countries (Argentina, Brazil, Chile, Colombia, Dominican Republic, Mexico, Paraguay, Peru, and the United States). Since the 2009 pandemic, 19 countries reached > 95% completeness with further improvements by 2019 when 24 countries reached > 95% completeness. This annual progression in influenza outcome surveillance illustrates the maturity of FluNet over time.

Jamaica, Paraguay, and Mexico were the first countries with available records starting in 2008 for both influenza A(H1N1)pdm09 and A(H5) (Fig. 1b,c). For A(H1N1)pdm09, completeness across the region reached ~ 70% by 2010 and slowly grew to ~ 80% by 2019. Some countries, including Uruguay, Ecuador and Nicaragua, show a reduction in completeness for A(H1N1)pdm09 after 2014. Some countries such as Suriname, Haiti, and Barbados did not offer surveillance data for A(H1N1)pdm09 until 2015. For influenza A(H5), completeness across countries almost reached 50% in 2010, then declined to 0% by 2016 and has not been reported by any country since. A similar trajectory was seen for specimens though this testing outcome continues to be reported by the United States and Canada as of 2019 (Fig. 1d).

The completeness of influenza A(H1) for the region grew from 43 to 70% between 2009 and 2011 and decreased gradually from 75% in 2014 to 56% as of 2019 (Fig. 2a). The completeness of influenza A(Unsubtyped) increased briefly in 2008 just prior to the A(H1N1)pdm09 pandemic. In 2009, the average completeness across countries increased from 46 to 75% completeness by 2011; it began to decline in 2014, surging back to ~ 70% completeness in 2017 but gradually declining again to 68% completeness as of 2019 (Fig. 2b).

Influenza B(Yamataga) and B(Victoria) subtypes have showed increased trends in completeness since 2012 (Fig. 2c,d). Both subtypes increased in completeness from 2007 to 2010 however the average completeness across countries did not exceed 50% for either subtype. After a brief decline from 2010 to 2011, the average completeness grew for both subtypes from 32% in 2012 to 75% in 2014. While declining from 2014 to 2016, completeness for these influenza B subtypes continuously increased thereafter and reached an average of ~ 67% across the region as of 2019.

In addition to temporal trends, we detected anomalies in annual completeness for influenza subtypes. For example, Fig. 1a showed that the United States, despite having 100% completeness in all other years, had only 63.5% completeness in 2006. During this year, we found that records for total positives are missing from Week 21 (22 May) to Week 49 (10 December). Yet, the United States Centers for Disease Control and Prevention (CDC) reported influenza records during these missing weeks^30^. Though cases are near zero, the national surveillance system does collect data on influenza positives that were not reported in FluNet.

Missing reports for influenza positives in 2018 from Week 30 (23 July) to Week 38 (23 September) in Peru provided another example of using annual completeness for spot-checking data quality (Fig. 1a). Reports from the Pan American Health Organization (PAHO) suggest that Peru reported a surge in influenza A(H1N1)pdm09 and SARI activity during these weeks^31–33^. Despite increased case counts, influenza activity dipped below the alert threshold with pneumonia cases increasing for infants < 5 years of age. Like in the United States, PAHO reported case information on influenza A positives, influenza B positives, and subtypes including A(H1N1)pdm09 and A(Unsubtyped) during these weeks^33^ though no data is reported within FluNet.

We also used annual completeness to identify patterns across variables and study years for specific countries. For example, while a surveillance system existed in Venezuela beginning in 2005, annual completeness varied greatly from 2005 to 2019 (Supplementary Table S5). Though surveillance data was reported for 2005–2007, completeness dropped to 0% for all influenza variables for 2008–2010 during the known rise of the A(H1N1)pdm09 pandemic. While 100% completeness was achieved for nearly all influenza outcomes and subtypes in 2011, completeness again dropped to 0% in 2012 and 2013. Since 2014, however, Venezuela maintained > 75% completeness for 5 of 6 influenza outcomes and 7 of 8 influenza subtypes. This fluctuation in annual completeness suggested that attributes related to the surveillance system or factors influencing surveillance performance such as economic stability and health expenditure could be influencing completeness.

### Overall completeness ranking and economic and health expenditure indicators

**Figure 3:**
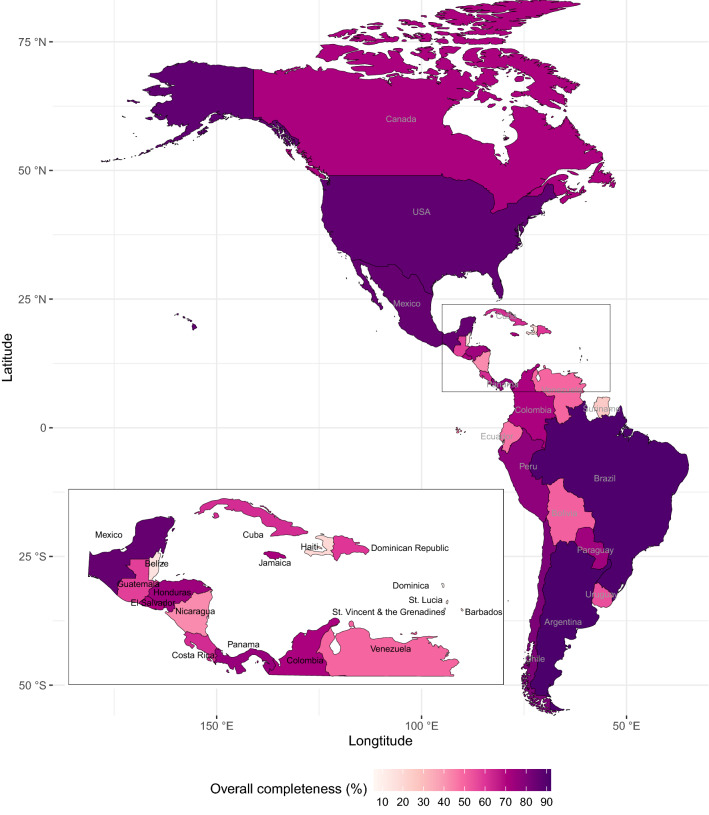
A map of average overall completeness for 29 Pan American countries over the 782-week time series starting from Week 1 (03 January) 2005 to Week 52 (29 December) 2019. A white color indicates near 0% completeness while a purple color indicates near 100% completeness.

**Figure 4:**
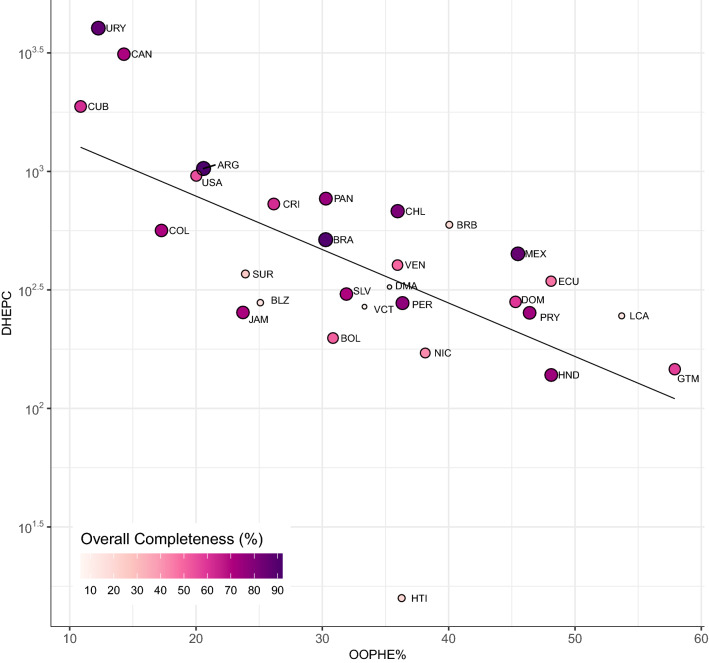
Relationship between overall completeness, domestic general government health expenditure per capita (DHEPC), and percent out-of-pocket health expenditure (OOPHE%) for 29 Pan American countries over the 782-week time series starting from Week 1 (03 January) 2005 to Week 52 (29 December) 2019. DHEPC is reported in current international USD. OOPHE% is reported as the percentage of current government expenditure. A smaller, white-colored marker indicates near 0% completeness while a larger, dark purple-colored marker indicates near 100% completeness.

The overall and average completeness values for six influenza outcomes for each country are compiled in Table 3 (all outcomes are reported in Supplementary Table S6). A map of average overall completeness is shown in Fig. 3. The overall completeness was > 80% for 4 countries (Argentina, Brazil, Mexico, United States) and > 70% for an additional 6 countries (Chile, Peru, El Salvador, Paraguay, Honduras, Panama). Completeness for tests, positives, influenza A positives, and influenza B positives were nearly identical for all countries except Brazil, Peru, Honduras, Jamaica, Costa Rica, Venezuela, and Ecuador where test completeness was slightly less than positives completeness. Eight countries (Argentina, Brazil, Mexico, United States, Chile, Peru, Colombia, and the Dominican Republic) had > 90% completeness for tests and total, influenza A, and influenza B positives. The average completeness values for influenza A(H1N1)pdm09 and A(H5) across all countries were 66% and 25%, respectively. Seven countries (Suriname, Haiti, Belize, Barbados, St. Lucia, Dominica, and St. Vincent & Grenadines) had < 40% completeness for all influenza outcomes, subtypes, and overall completeness. Figure 4 demonstrates that high and low overall completeness was likely in countries of mid-range DHEPC and OOPHE%. Given the similarity in completeness for multiple outcomes, we selected tests, positives, A(H1N1)pdm09, and overall completeness for further regression analyses.

**Table 3:** The overall and average completeness of seven influenza variables and three economic and health expenditure indicators for 2005–2019 time period for 29 Pan American countries and all countries combined.

Country	Percent completeness for influenza outcomes^a^							Indicators ^b^		
	Overall	Tests	Positives	Flu A	Flu B	A(H1N1)	Spec	GNIPC	DHEPC	OOPHE%
All countries	55.59	67.83	68.35	68.35	68.35	65.55	18.44	9.37	0.68	33.25
**Completeness > 75%**										
Argentina (ARG)	91.27	98.98	98.98	98.98	98.98	89.47	49.46	9.94	1.00	21.03
Brazil (BRA)	90.62	98.46	99.87	99.87	99.87	87.71	72.97	8.99	0.51	39.23
United States (USA)	86.91	97.56	97.56	97.56	97.56	89.47	91.67	52.67	4.67	12.37
Mexico (MEX)	85.83	99.87	99.87	99.87	99.87	91.67	0.00	9.39	0.43	44.72
Chile (CHL)	80.85	99.62	99.62	99.62	99.62	84.09	35.78	11.82	0.80	36.17
Peru (PER)	77.15	94.44	94.56	94.56	94.56	81.73	19.07	4.96	0.27	36.86
Panama (PAN)	75.03	81.64	81.64	81.64	81.64	83.49	33.33	9.11	0.75	30.02
**Completeness > 50%**										
Honduras (HND)	74.05	85.00	85.13	85.13	85.13	89.91	27.25	1.83	0.14	48.25
Paraguay (PRY)	73.46	88.46	88.46	88.46	88.46	92.15	11.22	4.21	0.24	43.76
Jamaica (JAM)	71.56	72.69	79.36	79.36	79.36	99.20	21.16	4.57	0.25	24.55
Colombia (COL)	71.07	98.08	98.08	98.08	98.08	90.87	0.00	5.83	0.45	20.63
Canada (CAN)	70.82	87.58	87.58	87.58	87.58	90.25	81.57	45.17	3.02	14.99
El Salvador (SLV)	70.69	80.92	80.92	80.92	80.92	89.77	45.46	3.12	0.30	32.70
Costa Rica (CRI)	62.17	78.28	80.33	80.33	80.33	83.17	7.05	8.29	0.72	26.72
Cuba (CUB)	61.92	72.70	72.70	72.70	72.70	90.87	2.56	5.87	1.85	10.90
Dominican Republic (DOM)	59.51	94.08	94.08	94.08	94.08	65.39	0.00	5.59	0.27	45.34
Guatemala (GTM)	57.88	67.52	67.52	67.52	67.52	78.96	16.93	3.08	0.14	58.77
Uruguay (URY)	54.69	76.48	76.48	76.48	76.48	69.18	2.52	11.51	0.93	20.32
Bolivia (BOL)	50.79	66.54	66.54	66.54	66.54	80.94	0.00	2.17	0.19	30.14
**Completeness < 50%**										
Venezuela (VEN)	49.63	55.27	57.71	57.71	57.71	56.91	13.99	10.01	0.32	38.66
Ecuador (ECU)	46.99	68.17	70.30	70.30	70.30	64.34	2.67	4.82	0.33	48.84
Nicaragua (NIC)	42.14	66.67	66.67	66.67	66.67	52.85	0.00	1.63	0.16	40.52
Suriname (SUR)	25.43	32.82	32.82	32.82	32.82	37.18	0.00	6.71	0.37	21.37
Haiti (HTI)	19.82	22.92	22.92	22.92	22.92	27.52	0.00	0.69	0.02	37.14
Barbados (BRB)	18.78	28.47	28.47	28.47	28.47	4.30	0.00	15.23	0.60	39.53
Belize (BLZ)	15.80	19.48	19.48	19.48	19.48	23.56	0.00	4.09	0.27	26.38
St. Lucia (LCA)	13.47	13.81	13.81	13.81	13.81	2.72	0.00	7.71	0.24	56.06
St. Vincent & the Grenadines (VCT)	7.51	6.28	6.28	6.28	6.28	1.28	0.00	6.27	0.26	21.68
Dominica (DMA)	6.17	14.29	14.29	14.29	14.29	1.91	0.00	6.53	0.32	36.65

Countries are listed in descending order by overall completeness.

^a^Outcomes: total specimens processed (*Test*), total positives (*Pos*), influenza A positives (*Flu A*), influenza B positives (*Flu B*), and total specimens collected (*Spec.*). For tests, total positives, influenza A and B positives, average completeness was calculated from 2005 to 2019. Subtypes of influenza positives: A(H1N1)pdm09 *(A(H1N1)))*. For A(H1N1)pdm09 and specimens collected, average completeness was calculated from 2008 to 2019. *Overall* provides the average completeness across all 14 influenza variables while *All Countries* provides the average completeness for each variable across all 29 countries.

^b^Reported values are the average of each indicator from 2005 to 2019. Units: GNIPC and DHEPC are provided in 1000 s USD. OOPHE% is reported as a percentage of current governmental health expenditure.

### Completeness, economic and health expenditure indicators, and surveillance system attributes

**Figure 5:**
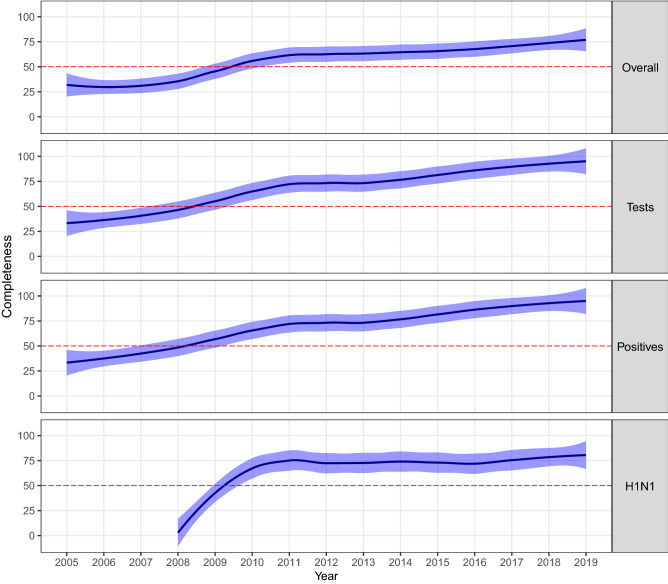
Annual trends presented as loess-smoothed curves along with their confidence intervals in country-specific completeness for four influenza outcome variables (overall, tests, positives, and A(H1N1)pdm09 completeness) for 29 Pan American countries over the 782-week time series starting from Week 1 (03 January) 2005 to Week 52 (29 December) 2019. Horizontal dashed red lines indicate 50% completeness.

**Figure 6:**
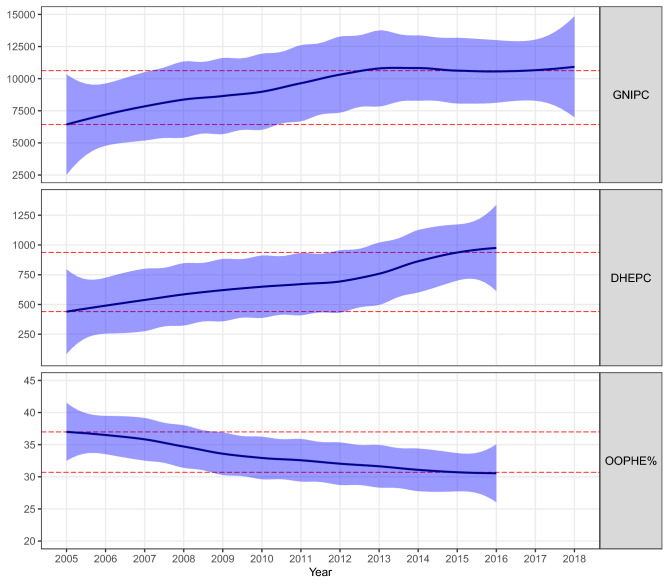
Annual trends presented as loess-smoothed curves along with their confidence intervals for three national economic and health expenditure indicators for 29 Pan American countries for available years between 2005 and 2018. Horizontal lines indicate regional average values for 2005 and 2015. GNIPC was reported in purchasing power parity (PPP) constant 2011 international US dollars (USD). DHEPC was reported in current international USD. OOPHE% is reported as the percentage of current government expenditure.

Trends in country-specific completeness for tests, positives, A(H1N1)pdm09, and overall and GNIPC, DHEPC, and OOPHE% are shown in Figs. 5 and 6, respectively. The decadal change of economic indicators for each country and averaged across countries (with standard deviation estimates) is provided in Supplementary Table S7. GNIPC and DHEPC almost doubled (2.02 ± 0.67 vs 2.19 ± 0.77, respectively) between 2005 and 2015; and OOPHE% declined by 16 ± 18%.

Table 4 shows the annual change in completeness for four outcome variables estimated from unadjusted (Model 1) and adjusted (Model 2) mixed effects models. All variables exhibited a strong positive trend with an annual increase in completeness from 4.2 to 6.3% on average. Improvements in completeness were most prominent for influenza A(H1N1)pdm09 showing a strong positive relationship with GNIPC. DHEPC was the strongest predictor of completeness across all influenza outcome variables. With the doubling in DHEPC achieved on average in the region, all four variables exhibited an improvement in completeness up to 9.4%. A projected 10% change in OOPHE% had no association with changes in completeness.

**Table 4:** Estimated annual change in completeness of four influenza outcome variables and three economic and health expenditure indicators in 29 Pan American countries over the 782-week time series starting from Week 1 (03 January) 2005 to Week 52 (29 December) 2019.

Outcome	Unadjusted estimates				Adjusted estimates^a^			
	Effect size	LCI	UCI	*p* value	Effect size	LCI	UCI	*p* value
**Estimated annual change in completeness**								
Overall	3.948	3.493	4.403	< 0.001	4.251	3.811	4.690	< 0.001
Tests	5.040	4.517	5.563	< 0.001	5.346	4.833	5.858	< 0.001
Positives	4.930	4.408	5.452	< 0.001	5.229	4.717	5.741	< 0.001
A(H1N1)pdm09	5.935	5.174	6.696	< 0.001	6.307	5.541	7.073	< 0.001
**Estimated effect associated with doubling in gross national income per capita**								
Overall	3.667	− 0.763	8.097	0.105	0.494	− 3.088	4.077	0.787
Tests	1.321	− 3.741	6.383	0.609	− 1.026	− 5.251	3.199	0.634
Positives	1.502	− 3.553	6.557	0.560	− 0.809	− 5.047	3.428	0.708
A(H1N1)pdm09	15.858	8.568	23.149	< 0.001	9.033	2.400	15.666	0.008
**Estimated effect associated with doubling in domestic health expenditure per capita**								
Overall	12.489	8.050	16.928	< 0.001	6.955	2.978	10.932	0.001
Tests	14.154	9.070	19.239	< 0.001	8.713	3.954	13.472	< 0.001
Positives	14.670	9.575	19.765	< 0.001	9.370	4.567	14.172	< 0.001
A(H1N1)pdm09	12.921	6.742	19.099	< 0.001	7.261	1.258	13.263	0.018
**Estimated effect associated with 10% change in out-of-pocket health expenditure**								
Overall	− 1.439	− 4.945	2.068	0.421	− 0.061	− 3.217	3.095	0.970
Tests	− 1.880	− 5.919	2.159	0.362	− 0.264	− 4.012	3.484	0.890
Positives	− 0.908	− 4.939	3.123	0.659	0.491	− 3.249	4.231	0.797
A(H1N1)pdm09	− 2.310	− 7.939	3.320	0.421	− 0.522	− 5.798	4.755	0.846

The effect size estimation was calculated for a doubling in per capita indicator estimates and a 10% increase in out-of-pocket health expenditure. *LCI* and *UCI* refer to the lower and upper bounds of the 95% confidence interval.

^a^Models were adjusted for case definition type, adherence to WHO definition, surveillance strategy, reporting quota, reporting timeframe, number of NICs and reporting facilities.

The adjusted models also indicated that the high numbers of NICS and reporting facilities were consistently associated with an increase in completeness. For every additional NIC, the completeness for tests, positives, and overall completeness increased by ~ 18–24% irrespective of the economic or expenditure indicator assessed. Similarly, every additional NIC was associated with a 15–20% increase in influenza A(H1N1)pdm09 completeness. A doubling in the number of in-country reporting facilities was associated with a 1.85–5.00% increase in completeness across all influenza outcomes and economic indicators. While some countries use multiple surveillance systems or followed numerous reporting quotas, timeframes, and case definitions, on average there was no difference in completeness values across surveillance system attributes.

## Discussion

In this study, we demonstrated the progress made by FluNet over the last decade towards achieving high overall completeness in publicly available influenza surveillance records. The proposed metric shows that influenza surveillance reporting has improved especially after the A(H1N1) 2009 pandemic, highlighting the effort by national systems and the WHO. This metric further demonstrated the substantial increase in completeness after the 2013 WHO guidelines for influenza surveillance^5^. These improvements were practically identical for tests, total positives, influenza A positives, and influenza B positives indicating the systematic approach taken in their reporting. As of 2019, 24 of 29 Pan-American countries were operating at > 95% of completeness for these key outcomes, though efforts are still needed to ensure consistent surveillance for other indicators. National reporting infrastructures continued to increase in their richness and heterogeneity, which indicates wider in-country surveillance coverage^14^. Annual completeness estimates continue to improve over time by ~ 5% annually and the rates of improvement are similar among countries with different national surveillance systems attributes. Yet, countries with higher numbers of NICs, more reporting facilities, and greater health expenditures showed the best performance. The proposed completeness metric provides essential information on data availability and suitability for statistical analysis and modeling and can improve the utility of existing data for all users.

Our metric is based on the effective time series length and can be computed for any pre-specified time periods. The metric ultimately reflects the fraction of weeks when surveillance reports are missing. We define ‘missing’ as a week for which case counts are undefined or not reported. This is not to be mistaken with zero reported observations, or the absence of counts for a specific case definition. To further the utility of the completeness metric, we recommend supplementing publicly disseminated reports with metadata on *why* data are missing, *what* missing data means, and *how much* missing data are reported.

Although the metric is simple to implement and interpret, the metric could not take into consideration patterns of missingness. Missing records could be distributed randomly or systematically throughout the study period when the data displays structural missingness with records lost in chunks or more frequently during specific times of the year. However, low completeness estimates call for attention to further investigate the pattern of missingness. This was demonstrated above in examples with the United States, Peru, and Venezuela where we identified periods of missing weeks and years using our metric. By knowing the temporal distribution of missing records some correction can be made during the analysis stage, for example by using completeness as an additional variable to reduce the weight of years with incomplete records. Closer inspection into patterns of missingness can also be used for verifying, inspecting, or updating public records within a data source prior to analysis.

The metric provides greater clarity on how a country’s surveillance system quality changes over time and helps identify anomalies in surveillance reporting. If an unusual drop in completeness is noted yet the national surveillance records exist for the time period in question, such discrepancies can be curated in a timely manner. Furthermore, such discrepancy calls into question whether external data users could help data curating organizations with checking the fidelity and accuracy of the data they use and their assumptions for handling missing data.

By examining annual completeness estimates, users could determine the study interval with reliable information. In the extracted records from 2005 to 2019, each country-outcome-specific time series had varying numbers and patterns of weeks with missing information. While a longer historical reference period indicates greater statistical power in estimating disease trends, some outcomes are collected over limited time frames. The length of these time series is influenced by the country of interest, outcome of interest, and date of data extraction. For example, positives of A(H1N1)pdm09 showed consistently high completeness across most countries from 2009 to 2019. Yet, for some countries, records were available for a fraction of that period. Based on our findings, we encourage data users to clearly specify the start and end date of their time series, the completeness of that time series, and the date of data extraction.

In recent years, the WHO has taken numerous efforts to evaluate the economic burden associated with seasonal influenza, especially in lower- and middle-income countries^34–36^. These efforts, including a published *Manual for estimating the economic burden of seasonal influenza* in 2016, aim to demonstrate the value of population surveillance by calculating the direct medical, direct non-medical, and indirect costs of influenza illness^34,35^. Studies applying methodologies outlined in this manual confirm that increased country income and health expenditure is associated with increased immunization policies, vaccination coverage, health infrastructure, and surveillance coverage^35,36^. The WHO 2016 manual further recognizes that data validity and completeness can influence assessments of economic burdens related to national income, domestic health expenditure, and out-of-pocket health expenditure^34^.

Our study faced several challenges related to data availability and accessibility. First, the FluNet data portal requires country time series to be downloaded individually and merged using a scripted pipeline. This process is both inefficient and prone to human error during data alignment and compilation. While our data extraction and merging code overcomes this challenge, we encourage FluNet curators to allow for multi-country data downloads and improve data accessibility.

Next, we examined only the most recent records starting in 2005. Our initial intention was to use all available data from 1995 to 2019. We completed two attempts to extract records: first on 15 December 2019 and second on 26 April 2020 to retrieve data for the final weeks of 2019. Between extraction dates, however, available FluNet data changed dramatically: data originally available from Week 1 1995 to Week 52 2004 were reported as missing at the time of second extraction. No justification is provided regarding this change. Thus, we encourage FluNet curators to provide information on when, by how much, and why retrospective records are modified to ensure accuracy and validity of analyses performed with the open source records.

Finally, we recognize that examined national economic indicators, such as DHEPC and OOPHE% describe all national health expenditures and the fraction of health expenditures dedicated to influenza may vary dramatically each year. During the A(H1N1)pdm09 pandemic, health expenditures may have been quite high for influenza vaccinations and testing. Moving forward, the onset of the coronavirus disease 2019 (COVID-19) pandemic may have decreased this fraction of health expenditures for influenza monitoring much lower. A better tracking of influenza-related expenditures at the national and global levels is needed to confirm or refute our findings.

In prior works, we have developed tools and explored the seasonality of influenza and other infections. In a study of pandemic seasonal epidemics in Wisconsin from 1967 to 2004, we found that seasonal peak timing varied greatly and while viral evolution played an important role, the variability of seasonality estimates was also influenced by the data granularity^23^. The estimates (and their confidence intervals) of seasonal peak timing and intensity could be in part influenced by data aggregation and completeness of surveillance data and thus affect our understanding of deviations in influenza seasonality^21^. Our mini-review of mathematical modeling techniques for influenza transmission highlighted that variations in theories governing seasonal dynamics could also be attributed to data availability limitations^22^.

Examining disease trends and seasonal epidemic signatures allows for greater understanding of influenza transmission and developing preparedness strategies at the local, national, and regional levels^2,18–20,36^. The proposed metric of completeness is essential in estimating the statistical power to detect disease trends and temporal changes by providing the effective length of disease surveillance time series data. Further work is needed to understand how completeness influences the reliability of modeling results. The use of the proposed metric will also allow for better assessment of the quality of historical data for tracking disease trends. The continuously updated surveillance records and the ensemble of disease outcomes allows for adaptive modeling to create real-time forecasts and detection of local events with high spatiotemporal granularity^2^.

## Conclusion

This study provided the first attempt at quantifying the percentage of available data usable for closer examination of disease trends or seasonality analyses. As more surveillance data becomes available for public use, indicators such as completeness should be applied to ensure quality, accuracy, and reliability of trend estimations. The proposed metric of completeness is vital to any secondary time series data analysis where the data user did not curate the data source. This metric can also be estimated and reported by external data users to ensure reliability and improve understanding of data structure. This next step in data sharing can help external data users detect outbreak signatures more accurately and reliably as well as improve health policies, programming, and recommendations. Combined with access to already developed WHO M&E indicators, the completeness metric for publicly disseminated data will strengthen disease surveillance systems.

## Supplementary Information

Supplementary Information.
