# Supplementary material for: Completeness of open access FluNet influenza surveillance data for Pan-America in 2005–2019
Source: Sci Rep. 2021 Jan 12;11:795. doi: 10.1038/s41598-020-80842-9 (PMC7804328; doi:10.1038/s41598-020-80842-9)
Supplement: Supplementary file 1 — Supplementary Information. [file 41598_2020_80842_MOESM1_ESM.docx]

Completeness of open access FluNet influenza surveillance data for Pan-America in 2005-2019

**Authors**

Ryan B. Simpson^1^, Jordyn Gottlieb^1^, Bingjie Zhou^1^, Meghan A. Hartwick^1^, and Elena N. Naumova^1^

**Affiliations**

^1^ Tufts University Friedman School of Nutrition Science and Policy

Corresponding Author(s): Elena N. Naumova (Elena.Naumova@tufts.edu)

**Supplementary Materials**

Supplementary Table S1. World Health Organization (WHO) influenza case definitions [6, 25-27]. 2

Supplementary Table S2. World Health Organization (WHO) surveillance strategies and reporting structures [6, 25-27]. 3

Supplementary Table S3. Economic and health expenditure indicators extracted from the World Bank's *World Development Indicators* publicly available database and their definitions [28]. 4

Supplementary Table S4. Annual completeness for 14 influenza variables from 2005-2019 for 29 Pan American countries. For 5 variables (A(H1N1)pdm09, A(H5), B(Yamagata), B(Victoria), and Specimens) average completeness values were corrected as shown in Table 2. 5

Supplementary Table S5. Annual completeness for 14 influenza variables from 2005-2019 for Venezuela. For 5 variables (A(H1N1)pdm09, A(H5), B(Yamagata), B(Victoria), and Specimens) average completeness values were corrected as shown in Table 2. 13

Supplementary Table S6. The average completeness of 14 influenza variables and overall from 2005-2019 for 29 Pan American countries and all countries combined. For 5 variables (A(H1N1)pdm09, A(H5), B(Yamagata), B(Victoria), and Specimens) average completeness values were corrected as shown in Table 2. 14

Supplementary Table S7. The decadal values and change (2005-2015) in three economic indicators (GNI per capita (GNIPC), domestic health expenditure per capita (DHEPC), and out-of-pocket health expenditure as a percentage of total health expenditure (OOPHE%)) for 29 Pan American countries. *Average* refers to the average decadal change across all countries. *Difference* refers to the percentage difference between 2005 and 2015 values. *Ratio* refers to the quotient of the 2015 and 2005 values.. 15

**Appendix.** R codes for data scraping and merging………………………………………………………...**16**

**Supplementary Table S1.** World Health Organization (WHO) influenza case definitions [6, 25-27].

| **WHO Case Definitions** |  |
| --- | --- |
| Influenza-like Illness (ILI) | An acute respiratory infection with: measured fever of ≥ 38 C°; and cough; with onset within the last 10 days. Identified in outpatient setting. |
| Pneumonia | Cough OR difficulty in breathing AND breathing faster than 40 breaths/minute (12 – 59 months); breathing faster than 50 breaths/minute (2 – 11 months); Severe pneumonia: cough OR difficulty in breathing AND any of the following general severe signs: unable to drink or breastfeed OR vomits everything OR convulsions OR lethargy or unconsciousness OR chest in drawing or stridor in a calm child. Requires management in outpatient setting. |
| Severe Acute Respiratory Infection (SARI) | An acute respiratory infection with: history of fever or measured fever of ≥ 38 C°; and cough; with onset within the last 10 days; and requires hospitalization. Identified in hospital setting. |
| Acute Respiratory Infection (ARI) | Sudden/acute onset of at least one of the following four respiratory symptoms: cough, sore throat, shortness of breath, coryza; and a clinician’s judgement that the illness is due to an infection. Identified in outpatient facility. |

**Supplementary Table S2.** World Health Organization (WHO) surveillance strategies and reporting structures [6, 25-27].

| **Surveillance Strategy and Reporting Structures** | |
| --- | --- |
| Sentinel Surveillance | Designated healthcare facilities/providers by a country’s Ministry of Health intended to be representative of the population. Sentinel consistently collect epidemiological information from patients that adhere to specific case definitions, usually ILI and SARI. Samples are taken from randomized/standardized protocols. |
| Universal Surveillance | All designated healthcare facilities and providers; includes reporting of all clinician-defined ARI and part of national disease surveillance system. Purpose is to provide understanding of clinician-reported respiratory disease prevalence. |
| National Surveillance | All healthcare facilities not in selected sentinel sites. Monitors unusual and unexpected SARI cases and mortalities. |
| National Influenza Center (NIC) | Nationally recognized institutions approved by WHO officials to be in compliance with WHO terms of reference (TORs) for influenza surveillance on an annual basis). A NIC may have additional obligations under the national authorities of its respective country. WHO will generally be recognizing no more than one NIC per country. |
| Reporting Facilities | Reporting facilities include SARI hospitals, ILI centers, polymer chain reaction (PCR) testing facilities, and influenza (IF) testing laboratories. |

**Supplementary Table S3.** Economic and health expenditure indicators extracted from the World Bank's *World Development Indicators* publicly available database and their definitions [28].

| **Indicator** | **Definition** |
| --- | --- |
| GNI per capita, PPP (constant 2011 international $) (GNIPC) | GNI per capita based on purchasing power parity (PPP). PPP GNI is gross national income (GNI) converted to international dollars using purchasing power parity rates. An international dollar has the same purchasing power over GNI as a U.S. dollar has in the United States. GNI is the sum of value added by all resident producers plus any product taxes (less subsidies) not included in the valuation of output plus net receipts of primary income (compensation of employees and property income) from abroad. Data are in constant 2011 international dollars. |
| Domestic general government health expenditure per capita, PPP (current international $)  (DHEPC) | Public expenditure on health from domestic sources per capita expressed in international dollars at purchasing power parity (PPP). |
| Out-of-pocket health expenditure (% current expenditure) (OOPHE%) | Share of out-of-pocket payments of total current health expenditures. Out-of-pocket payments are spending on health directly out-of-pocket by households. |

**Supplementary Table S4.** Annual completeness for 14 influenza variables from 2005-2019 for 29 Pan American countries. For 5 variables (A(H1N1)pdm09, A(H5), B(Yamagata), B(Victoria), and Specimens) average completeness values were corrected as shown in Table 2.

| Country | 2005 | 2006 | 2007 | 2008 | 2009 | 2010 | 2011 | 2012 | 2013 | 2014 | 2015 | 2016 | 2017 | 2018 | 2019 | Average |
| --- | --- | --- | --- | --- | --- | --- | --- | --- | --- | --- | --- | --- | --- | --- | --- | --- |
| **Tests** | | | | | | | | | | | | | | | | |
| Argentina | 98.08 | 100.00 | 94.23 | 96.15 | 96.23 | 100.00 | 100.00 | 100.00 | 100.00 | 100.00 | 100.00 | 100.00 | 100.00 | 100.00 | 100.00 | 98.98 |
| Barbados | 0.00 | 0.00 | 0.00 | 0.00 | 0.00 | 0.00 | 0.00 | 0.00 | 0.00 | 0.00 | 92.45 | 88.46 | 80.77 | 84.62 | 80.77 | 28.47 |
| Belize | 0.00 | 0.00 | 0.00 | 0.00 | 0.00 | 0.00 | 0.00 | 0.00 | 0.00 | 0.00 | 5.66 | 0.00 | 98.08 | 88.46 | 100.00 | 19.48 |
| Bolivia | 0.00 | 0.00 | 0.00 | 0.00 | 0.00 | 100.00 | 100.00 | 100.00 | 100.00 | 100.00 | 98.11 | 100.00 | 100.00 | 100.00 | 100.00 | 66.54 |
| Brazil | 100.00 | 100.00 | 100.00 | 100.00 | 100.00 | 100.00 | 100.00 | 100.00 | 100.00 | 100.00 | 100.00 | 78.85 | 100.00 | 100.00 | 98.08 | 98.46 |
| Canada | 0.00 | 34.62 | 96.15 | 100.00 | 86.79 | 100.00 | 98.08 | 98.08 | 100.00 | 100.00 | 100.00 | 100.00 | 100.00 | 100.00 | 100.00 | 87.58 |
| Chile | 100.00 | 100.00 | 98.08 | 100.00 | 96.23 | 100.00 | 100.00 | 100.00 | 100.00 | 100.00 | 100.00 | 100.00 | 100.00 | 100.00 | 100.00 | 99.62 |
| Colombia | 100.00 | 100.00 | 100.00 | 80.77 | 98.11 | 100.00 | 100.00 | 100.00 | 96.15 | 100.00 | 96.23 | 100.00 | 100.00 | 100.00 | 100.00 | 98.08 |
| Costa Rica | 5.77 | 9.62 | 40.38 | 80.77 | 41.51 | 100.00 | 100.00 | 100.00 | 100.00 | 100.00 | 98.11 | 100.00 | 100.00 | 98.08 | 100.00 | 78.28 |
| Cuba | 0.00 | 0.00 | 0.00 | 0.00 | 98.11 | 100.00 | 100.00 | 98.08 | 100.00 | 100.00 | 98.11 | 100.00 | 98.08 | 100.00 | 98.08 | 72.70 |
| Dominica | 0.00 | 0.00 | 0.00 | 0.00 | 0.00 | 0.00 | 0.00 | 0.00 | 0.00 | 0.00 | 52.83 | 0.00 | 42.31 | 55.77 | 63.46 | 14.29 |
| Dominican Republic | 100.00 | 100.00 | 100.00 | 100.00 | 16.98 | 100.00 | 100.00 | 100.00 | 100.00 | 96.15 | 98.11 | 100.00 | 100.00 | 100.00 | 100.00 | 94.08 |
| Ecuador | 0.00 | 0.00 | 3.85 | 0.00 | 32.08 | 88.46 | 100.00 | 100.00 | 100.00 | 100.00 | 98.11 | 100.00 | 100.00 | 100.00 | 100.00 | 68.17 |
| El Salvador | 0.00 | 82.69 | 0.00 | 53.85 | 84.91 | 96.15 | 100.00 | 100.00 | 100.00 | 100.00 | 100.00 | 100.00 | 100.00 | 96.15 | 100.00 | 80.92 |
| Guatemala | 0.00 | 0.00 | 0.00 | 0.00 | 33.96 | 98.08 | 100.00 | 96.15 | 100.00 | 94.23 | 98.11 | 100.00 | 92.31 | 100.00 | 100.00 | 67.52 |
| Haiti | 0.00 | 0.00 | 0.00 | 0.00 | 0.00 | 0.00 | 0.00 | 0.00 | 0.00 | 0.00 | 24.53 | 26.92 | 100.00 | 100.00 | 92.31 | 22.92 |
| Honduras | 0.00 | 0.00 | 100.00 | 96.15 | 98.11 | 100.00 | 100.00 | 96.15 | 98.08 | 92.31 | 98.11 | 100.00 | 98.08 | 100.00 | 98.08 | 85.00 |
| Jamaica | 0.00 | 0.00 | 0.00 | 0.00 | 98.11 | 100.00 | 98.08 | 100.00 | 100.00 | 100.00 | 100.00 | 96.15 | 100.00 | 98.08 | 100.00 | 72.69 |
| Mexico | 100.00 | 100.00 | 100.00 | 100.00 | 100.00 | 100.00 | 100.00 | 100.00 | 100.00 | 100.00 | 98.11 | 100.00 | 100.00 | 100.00 | 100.00 | 99.87 |
| Nicaragua | 0.00 | 0.00 | 0.00 | 0.00 | 0.00 | 100.00 | 100.00 | 100.00 | 100.00 | 100.00 | 100.00 | 100.00 | 100.00 | 100.00 | 100.00 | 66.67 |
| Panama | 0.00 | 0.00 | 100.00 | 100.00 | 24.53 | 100.00 | 100.00 | 100.00 | 100.00 | 100.00 | 100.00 | 100.00 | 100.00 | 100.00 | 100.00 | 81.64 |
| Paraguay | 100.00 | 30.77 | 0.00 | 98.08 | 100.00 | 100.00 | 100.00 | 100.00 | 100.00 | 100.00 | 98.11 | 100.00 | 100.00 | 100.00 | 100.00 | 88.46 |
| Peru | 100.00 | 100.00 | 100.00 | 98.08 | 37.74 | 100.00 | 100.00 | 100.00 | 100.00 | 100.00 | 100.00 | 98.08 | 100.00 | 82.69 | 100.00 | 94.44 |
| St. Lucia | 0.00 | 0.00 | 0.00 | 0.00 | 0.00 | 0.00 | 0.00 | 0.00 | 0.00 | 0.00 | 26.42 | 0.00 | 44.23 | 73.08 | 63.46 | 13.81 |
| St. Vincent & the Grenadines | 0.00 | 0.00 | 0.00 | 0.00 | 0.00 | 0.00 | 0.00 | 0.00 | 0.00 | 0.00 | 3.77 | 0.00 | 3.85 | 21.15 | 65.38 | 6.28 |
| Suriname | 0.00 | 0.00 | 0.00 | 0.00 | 0.00 | 0.00 | 0.00 | 0.00 | 0.00 | 0.00 | 98.11 | 100.00 | 100.00 | 98.08 | 96.15 | 32.82 |
| United States | 100.00 | 63.46 | 100.00 | 100.00 | 100.00 | 100.00 | 100.00 | 100.00 | 100.00 | 100.00 | 100.00 | 100.00 | 100.00 | 100.00 | 100.00 | 97.56 |
| Uruguay | 51.92 | 46.15 | 63.46 | 59.62 | 47.17 | 50.00 | 71.15 | 75.00 | 100.00 | 100.00 | 98.11 | 100.00 | 96.15 | 90.38 | 98.08 | 76.48 |
| Venezuela | 3.85 | 42.31 | 100.00 | 0.00 | 0.00 | 0.00 | 100.00 | 0.00 | 0.00 | 100.00 | 88.68 | 100.00 | 98.08 | 98.08 | 98.08 | 55.27 |
| **Average** | **33.09** | **34.81** | **44.69** | **47.02** | **47.95** | **70.09** | **74.73** | **71.15** | **72.21** | **75.27** | **85.17** | **82.36** | **91.45** | **92.57** | **94.89** | **67.83** |

| **Positives** | | | | | | | | | | | | | | | | |
| --- | --- | --- | --- | --- | --- | --- | --- | --- | --- | --- | --- | --- | --- | --- | --- | --- |
| Argentina | 98.08 | 100.00 | 94.23 | 96.15 | 96.23 | 100.00 | 100.00 | 100.00 | 100.00 | 100.00 | 100.00 | 100.00 | 100.00 | 100.00 | 100.00 | 98.98 |
| Barbados | 0.00 | 0.00 | 0.00 | 0.00 | 0.00 | 0.00 | 0.00 | 0.00 | 0.00 | 0.00 | 92.45 | 88.46 | 80.77 | 84.62 | 80.77 | 28.47 |
| Belize | 0.00 | 0.00 | 0.00 | 0.00 | 0.00 | 0.00 | 0.00 | 0.00 | 0.00 | 0.00 | 5.66 | 0.00 | 98.08 | 88.46 | 100.00 | 19.48 |
| Bolivia | 0.00 | 0.00 | 0.00 | 0.00 | 0.00 | 100.00 | 100.00 | 100.00 | 100.00 | 100.00 | 98.11 | 100.00 | 100.00 | 100.00 | 100.00 | 66.54 |
| Brazil | 100.00 | 100.00 | 100.00 | 100.00 | 100.00 | 100.00 | 100.00 | 100.00 | 100.00 | 100.00 | 100.00 | 100.00 | 100.00 | 100.00 | 98.08 | 99.87 |
| Canada | 0.00 | 34.62 | 96.15 | 100.00 | 86.79 | 100.00 | 98.08 | 98.08 | 100.00 | 100.00 | 100.00 | 100.00 | 100.00 | 100.00 | 100.00 | 87.58 |
| Chile | 100.00 | 100.00 | 98.08 | 100.00 | 96.23 | 100.00 | 100.00 | 100.00 | 100.00 | 100.00 | 100.00 | 100.00 | 100.00 | 100.00 | 100.00 | 99.62 |
| Colombia | 100.00 | 100.00 | 100.00 | 80.77 | 98.11 | 100.00 | 100.00 | 100.00 | 96.15 | 100.00 | 96.23 | 100.00 | 100.00 | 100.00 | 100.00 | 98.08 |
| Costa Rica | 5.77 | 21.15 | 40.38 | 100.00 | 41.51 | 100.00 | 100.00 | 100.00 | 100.00 | 100.00 | 98.11 | 100.00 | 100.00 | 98.08 | 100.00 | 80.33 |
| Cuba | 0.00 | 0.00 | 0.00 | 0.00 | 98.11 | 100.00 | 100.00 | 98.08 | 100.00 | 100.00 | 98.11 | 100.00 | 98.08 | 100.00 | 98.08 | 72.70 |
| Dominica | 0.00 | 0.00 | 0.00 | 0.00 | 0.00 | 0.00 | 0.00 | 0.00 | 0.00 | 0.00 | 52.83 | 0.00 | 42.31 | 55.77 | 63.46 | 14.29 |
| Dominican Republic | 100.00 | 100.00 | 100.00 | 100.00 | 16.98 | 100.00 | 100.00 | 100.00 | 100.00 | 96.15 | 98.11 | 100.00 | 100.00 | 100.00 | 100.00 | 94.08 |
| Ecuador | 0.00 | 0.00 | 3.85 | 0.00 | 64.15 | 88.46 | 100.00 | 100.00 | 100.00 | 100.00 | 98.11 | 100.00 | 100.00 | 100.00 | 100.00 | 70.30 |
| El Salvador | 0.00 | 82.69 | 0.00 | 53.85 | 84.91 | 96.15 | 100.00 | 100.00 | 100.00 | 100.00 | 100.00 | 100.00 | 100.00 | 96.15 | 100.00 | 80.92 |
| Guatemala | 0.00 | 0.00 | 0.00 | 0.00 | 33.96 | 98.08 | 100.00 | 96.15 | 100.00 | 94.23 | 98.11 | 100.00 | 92.31 | 100.00 | 100.00 | 67.52 |
| Haiti | 0.00 | 0.00 | 0.00 | 0.00 | 0.00 | 0.00 | 0.00 | 0.00 | 0.00 | 0.00 | 24.53 | 26.92 | 100.00 | 100.00 | 92.31 | 22.92 |
| Honduras | 0.00 | 0.00 | 100.00 | 98.08 | 98.11 | 100.00 | 100.00 | 96.15 | 98.08 | 92.31 | 98.11 | 100.00 | 98.08 | 100.00 | 98.08 | 85.13 |
| Jamaica | 0.00 | 0.00 | 0.00 | 100.00 | 98.11 | 100.00 | 98.08 | 100.00 | 100.00 | 100.00 | 100.00 | 96.15 | 100.00 | 98.08 | 100.00 | 79.36 |
| Mexico | 100.00 | 100.00 | 100.00 | 100.00 | 100.00 | 100.00 | 100.00 | 100.00 | 100.00 | 100.00 | 98.11 | 100.00 | 100.00 | 100.00 | 100.00 | 99.87 |
| Nicaragua | 0.00 | 0.00 | 0.00 | 0.00 | 0.00 | 100.00 | 100.00 | 100.00 | 100.00 | 100.00 | 100.00 | 100.00 | 100.00 | 100.00 | 100.00 | 66.67 |
| Panama | 0.00 | 0.00 | 100.00 | 100.00 | 24.53 | 100.00 | 100.00 | 100.00 | 100.00 | 100.00 | 100.00 | 100.00 | 100.00 | 100.00 | 100.00 | 81.64 |
| Paraguay | 100.00 | 30.77 | 0.00 | 98.08 | 100.00 | 100.00 | 100.00 | 100.00 | 100.00 | 100.00 | 98.11 | 100.00 | 100.00 | 100.00 | 100.00 | 88.46 |
| Peru | 100.00 | 100.00 | 100.00 | 98.08 | 39.62 | 100.00 | 100.00 | 100.00 | 100.00 | 100.00 | 100.00 | 98.08 | 100.00 | 82.69 | 100.00 | 94.56 |
| St. Lucia | 0.00 | 0.00 | 0.00 | 0.00 | 0.00 | 0.00 | 0.00 | 0.00 | 0.00 | 0.00 | 26.42 | 0.00 | 44.23 | 73.08 | 63.46 | 13.81 |
| St. Vincent & the Grenadines | 0.00 | 0.00 | 0.00 | 0.00 | 0.00 | 0.00 | 0.00 | 0.00 | 0.00 | 0.00 | 3.77 | 0.00 | 3.85 | 21.15 | 65.38 | 6.28 |
| Suriname | 0.00 | 0.00 | 0.00 | 0.00 | 0.00 | 0.00 | 0.00 | 0.00 | 0.00 | 0.00 | 98.11 | 100.00 | 100.00 | 98.08 | 96.15 | 32.82 |
| United States | 100.00 | 63.46 | 100.00 | 100.00 | 100.00 | 100.00 | 100.00 | 100.00 | 100.00 | 100.00 | 100.00 | 100.00 | 100.00 | 100.00 | 100.00 | 97.56 |
| Uruguay | 51.92 | 46.15 | 63.46 | 59.62 | 47.17 | 50.00 | 71.15 | 75.00 | 100.00 | 100.00 | 98.11 | 100.00 | 96.15 | 90.38 | 98.08 | 76.48 |
| Venezuela | 15.38 | 67.31 | 100.00 | 0.00 | 0.00 | 0.00 | 100.00 | 0.00 | 0.00 | 100.00 | 88.68 | 100.00 | 98.08 | 98.08 | 98.08 | 57.71 |
| **Average** | **33.49** | **36.07** | **44.69** | **51.19** | **49.12** | **70.09** | **74.73** | **71.15** | **72.21** | **75.27** | **85.17** | **83.09** | **91.45** | **92.57** | **94.89** | **68.35** |

| **Influenza A Positives** | | | | | | | | | | | | | | | | |
| --- | --- | --- | --- | --- | --- | --- | --- | --- | --- | --- | --- | --- | --- | --- | --- | --- |
| Argentina | 98.08 | 100.00 | 94.23 | 96.15 | 96.23 | 100.00 | 100.00 | 100.00 | 100.00 | 100.00 | 100.00 | 100.00 | 100.00 | 100.00 | 100.00 | 98.98 |
| Barbados | 0.00 | 0.00 | 0.00 | 0.00 | 0.00 | 0.00 | 0.00 | 0.00 | 0.00 | 0.00 | 92.45 | 88.46 | 80.77 | 84.62 | 80.77 | 28.47 |
| Belize | 0.00 | 0.00 | 0.00 | 0.00 | 0.00 | 0.00 | 0.00 | 0.00 | 0.00 | 0.00 | 5.66 | 0.00 | 98.08 | 88.46 | 100.00 | 19.48 |
| Bolivia | 0.00 | 0.00 | 0.00 | 0.00 | 0.00 | 100.00 | 100.00 | 100.00 | 100.00 | 100.00 | 98.11 | 100.00 | 100.00 | 100.00 | 100.00 | 66.54 |
| Brazil | 100.00 | 100.00 | 100.00 | 100.00 | 100.00 | 100.00 | 100.00 | 100.00 | 100.00 | 100.00 | 100.00 | 100.00 | 100.00 | 100.00 | 98.08 | 99.87 |
| Canada | 0.00 | 34.62 | 96.15 | 100.00 | 86.79 | 100.00 | 98.08 | 98.08 | 100.00 | 100.00 | 100.00 | 100.00 | 100.00 | 100.00 | 100.00 | 87.58 |
| Chile | 100.00 | 100.00 | 98.08 | 100.00 | 96.23 | 100.00 | 100.00 | 100.00 | 100.00 | 100.00 | 100.00 | 100.00 | 100.00 | 100.00 | 100.00 | 99.62 |
| Colombia | 100.00 | 100.00 | 100.00 | 80.77 | 98.11 | 100.00 | 100.00 | 100.00 | 96.15 | 100.00 | 96.23 | 100.00 | 100.00 | 100.00 | 100.00 | 98.08 |
| Costa Rica | 5.77 | 21.15 | 40.38 | 100.00 | 41.51 | 100.00 | 100.00 | 100.00 | 100.00 | 100.00 | 98.11 | 100.00 | 100.00 | 98.08 | 100.00 | 80.33 |
| Cuba | 0.00 | 0.00 | 0.00 | 0.00 | 98.11 | 100.00 | 100.00 | 98.08 | 100.00 | 100.00 | 98.11 | 100.00 | 98.08 | 100.00 | 98.08 | 72.70 |
| Dominica | 0.00 | 0.00 | 0.00 | 0.00 | 0.00 | 0.00 | 0.00 | 0.00 | 0.00 | 0.00 | 52.83 | 0.00 | 42.31 | 55.77 | 63.46 | 14.29 |
| Dominican Republic | 100.00 | 100.00 | 100.00 | 100.00 | 16.98 | 100.00 | 100.00 | 100.00 | 100.00 | 96.15 | 98.11 | 100.00 | 100.00 | 100.00 | 100.00 | 94.08 |
| Ecuador | 0.00 | 0.00 | 3.85 | 0.00 | 64.15 | 88.46 | 100.00 | 100.00 | 100.00 | 100.00 | 98.11 | 100.00 | 100.00 | 100.00 | 100.00 | 70.30 |
| El Salvador | 0.00 | 82.69 | 0.00 | 53.85 | 84.91 | 96.15 | 100.00 | 100.00 | 100.00 | 100.00 | 100.00 | 100.00 | 100.00 | 96.15 | 100.00 | 80.92 |
| Guatemala | 0.00 | 0.00 | 0.00 | 0.00 | 33.96 | 98.08 | 100.00 | 96.15 | 100.00 | 94.23 | 98.11 | 100.00 | 92.31 | 100.00 | 100.00 | 67.52 |
| Haiti | 0.00 | 0.00 | 0.00 | 0.00 | 0.00 | 0.00 | 0.00 | 0.00 | 0.00 | 0.00 | 24.53 | 26.92 | 100.00 | 100.00 | 92.31 | 22.92 |
| Honduras | 0.00 | 0.00 | 100.00 | 98.08 | 98.11 | 100.00 | 100.00 | 96.15 | 98.08 | 92.31 | 98.11 | 100.00 | 98.08 | 100.00 | 98.08 | 85.13 |
| Jamaica | 0.00 | 0.00 | 0.00 | 100.00 | 98.11 | 100.00 | 98.08 | 100.00 | 100.00 | 100.00 | 100.00 | 96.15 | 100.00 | 98.08 | 100.00 | 79.36 |
| Mexico | 100.00 | 100.00 | 100.00 | 100.00 | 100.00 | 100.00 | 100.00 | 100.00 | 100.00 | 100.00 | 98.11 | 100.00 | 100.00 | 100.00 | 100.00 | 99.87 |
| Nicaragua | 0.00 | 0.00 | 0.00 | 0.00 | 0.00 | 100.00 | 100.00 | 100.00 | 100.00 | 100.00 | 100.00 | 100.00 | 100.00 | 100.00 | 100.00 | 66.67 |
| Panama | 0.00 | 0.00 | 100.00 | 100.00 | 24.53 | 100.00 | 100.00 | 100.00 | 100.00 | 100.00 | 100.00 | 100.00 | 100.00 | 100.00 | 100.00 | 81.64 |
| Paraguay | 100.00 | 30.77 | 0.00 | 98.08 | 100.00 | 100.00 | 100.00 | 100.00 | 100.00 | 100.00 | 98.11 | 100.00 | 100.00 | 100.00 | 100.00 | 88.46 |
| Peru | 100.00 | 100.00 | 100.00 | 98.08 | 39.62 | 100.00 | 100.00 | 100.00 | 100.00 | 100.00 | 100.00 | 98.08 | 100.00 | 82.69 | 100.00 | 94.56 |
| St. Lucia | 0.00 | 0.00 | 0.00 | 0.00 | 0.00 | 0.00 | 0.00 | 0.00 | 0.00 | 0.00 | 26.42 | 0.00 | 44.23 | 73.08 | 63.46 | 13.81 |
| St. Vincent & the Grenadines | 0.00 | 0.00 | 0.00 | 0.00 | 0.00 | 0.00 | 0.00 | 0.00 | 0.00 | 0.00 | 3.77 | 0.00 | 3.85 | 21.15 | 65.38 | 6.28 |
| Suriname | 0.00 | 0.00 | 0.00 | 0.00 | 0.00 | 0.00 | 0.00 | 0.00 | 0.00 | 0.00 | 98.11 | 100.00 | 100.00 | 98.08 | 96.15 | 32.82 |
| United States | 100.00 | 63.46 | 100.00 | 100.00 | 100.00 | 100.00 | 100.00 | 100.00 | 100.00 | 100.00 | 100.00 | 100.00 | 100.00 | 100.00 | 100.00 | 97.56 |
| Uruguay | 51.92 | 46.15 | 63.46 | 59.62 | 47.17 | 50.00 | 71.15 | 75.00 | 100.00 | 100.00 | 98.11 | 100.00 | 96.15 | 90.38 | 98.08 | 76.48 |
| Venezuela | 15.38 | 67.31 | 100.00 | 0.00 | 0.00 | 0.00 | 100.00 | 0.00 | 0.00 | 100.00 | 88.68 | 100.00 | 98.08 | 98.08 | 98.08 | 57.71 |
| **Average** | **33.49** | **36.07** | **44.69** | **51.19** | **49.12** | **70.09** | **74.73** | **71.15** | **72.21** | **75.27** | **85.17** | **83.09** | **91.45** | **92.57** | **94.89** | **68.35** |

| **Influenza B Positives** | | | | | | | | | | | | | | | | |
| --- | --- | --- | --- | --- | --- | --- | --- | --- | --- | --- | --- | --- | --- | --- | --- | --- |
| Argentina | 98.08 | 100.00 | 94.23 | 96.15 | 96.23 | 100.00 | 100.00 | 100.00 | 100.00 | 100.00 | 100.00 | 100.00 | 100.00 | 100.00 | 100.00 | 98.98 |
| Barbados | 0.00 | 0.00 | 0.00 | 0.00 | 0.00 | 0.00 | 0.00 | 0.00 | 0.00 | 0.00 | 92.45 | 88.46 | 80.77 | 84.62 | 80.77 | 28.47 |
| Belize | 0.00 | 0.00 | 0.00 | 0.00 | 0.00 | 0.00 | 0.00 | 0.00 | 0.00 | 0.00 | 5.66 | 0.00 | 98.08 | 88.46 | 100.00 | 19.48 |
| Bolivia | 0.00 | 0.00 | 0.00 | 0.00 | 0.00 | 100.00 | 100.00 | 100.00 | 100.00 | 100.00 | 98.11 | 100.00 | 100.00 | 100.00 | 100.00 | 66.54 |
| Brazil | 100.00 | 100.00 | 100.00 | 100.00 | 100.00 | 100.00 | 100.00 | 100.00 | 100.00 | 100.00 | 100.00 | 100.00 | 100.00 | 100.00 | 98.08 | 99.87 |
| Canada | 0.00 | 34.62 | 96.15 | 100.00 | 86.79 | 100.00 | 98.08 | 98.08 | 100.00 | 100.00 | 100.00 | 100.00 | 100.00 | 100.00 | 100.00 | 87.58 |
| Chile | 100.00 | 100.00 | 98.08 | 100.00 | 96.23 | 100.00 | 100.00 | 100.00 | 100.00 | 100.00 | 100.00 | 100.00 | 100.00 | 100.00 | 100.00 | 99.62 |
| Colombia | 100.00 | 100.00 | 100.00 | 80.77 | 98.11 | 100.00 | 100.00 | 100.00 | 96.15 | 100.00 | 96.23 | 100.00 | 100.00 | 100.00 | 100.00 | 98.08 |
| Costa Rica | 5.77 | 21.15 | 40.38 | 100.00 | 41.51 | 100.00 | 100.00 | 100.00 | 100.00 | 100.00 | 98.11 | 100.00 | 100.00 | 98.08 | 100.00 | 80.33 |
| Cuba | 0.00 | 0.00 | 0.00 | 0.00 | 98.11 | 100.00 | 100.00 | 98.08 | 100.00 | 100.00 | 98.11 | 100.00 | 98.08 | 100.00 | 98.08 | 72.70 |
| Dominica | 0.00 | 0.00 | 0.00 | 0.00 | 0.00 | 0.00 | 0.00 | 0.00 | 0.00 | 0.00 | 52.83 | 0.00 | 42.31 | 55.77 | 63.46 | 14.29 |
| Dominican Republic | 100.00 | 100.00 | 100.00 | 100.00 | 16.98 | 100.00 | 100.00 | 100.00 | 100.00 | 96.15 | 98.11 | 100.00 | 100.00 | 100.00 | 100.00 | 94.08 |
| Ecuador | 0.00 | 0.00 | 3.85 | 0.00 | 64.15 | 88.46 | 100.00 | 100.00 | 100.00 | 100.00 | 98.11 | 100.00 | 100.00 | 100.00 | 100.00 | 70.30 |
| El Salvador | 0.00 | 82.69 | 0.00 | 53.85 | 84.91 | 96.15 | 100.00 | 100.00 | 100.00 | 100.00 | 100.00 | 100.00 | 100.00 | 96.15 | 100.00 | 80.92 |
| Guatemala | 0.00 | 0.00 | 0.00 | 0.00 | 33.96 | 98.08 | 100.00 | 96.15 | 100.00 | 94.23 | 98.11 | 100.00 | 92.31 | 100.00 | 100.00 | 67.52 |
| Haiti | 0.00 | 0.00 | 0.00 | 0.00 | 0.00 | 0.00 | 0.00 | 0.00 | 0.00 | 0.00 | 24.53 | 26.92 | 100.00 | 100.00 | 92.31 | 22.92 |
| Honduras | 0.00 | 0.00 | 100.00 | 98.08 | 98.11 | 100.00 | 100.00 | 96.15 | 98.08 | 92.31 | 98.11 | 100.00 | 98.08 | 100.00 | 98.08 | 85.13 |
| Jamaica | 0.00 | 0.00 | 0.00 | 100.00 | 98.11 | 100.00 | 98.08 | 100.00 | 100.00 | 100.00 | 100.00 | 96.15 | 100.00 | 98.08 | 100.00 | 79.36 |
| Mexico | 100.00 | 100.00 | 100.00 | 100.00 | 100.00 | 100.00 | 100.00 | 100.00 | 100.00 | 100.00 | 98.11 | 100.00 | 100.00 | 100.00 | 100.00 | 99.87 |
| Nicaragua | 0.00 | 0.00 | 0.00 | 0.00 | 0.00 | 100.00 | 100.00 | 100.00 | 100.00 | 100.00 | 100.00 | 100.00 | 100.00 | 100.00 | 100.00 | 66.67 |
| Panama | 0.00 | 0.00 | 100.00 | 100.00 | 24.53 | 100.00 | 100.00 | 100.00 | 100.00 | 100.00 | 100.00 | 100.00 | 100.00 | 100.00 | 100.00 | 81.64 |
| Paraguay | 100.00 | 30.77 | 0.00 | 98.08 | 100.00 | 100.00 | 100.00 | 100.00 | 100.00 | 100.00 | 98.11 | 100.00 | 100.00 | 100.00 | 100.00 | 88.46 |
| Peru | 100.00 | 100.00 | 100.00 | 98.08 | 39.62 | 100.00 | 100.00 | 100.00 | 100.00 | 100.00 | 100.00 | 98.08 | 100.00 | 82.69 | 100.00 | 94.56 |
| St. Lucia | 0.00 | 0.00 | 0.00 | 0.00 | 0.00 | 0.00 | 0.00 | 0.00 | 0.00 | 0.00 | 26.42 | 0.00 | 44.23 | 73.08 | 63.46 | 13.81 |
| St. Vincent & the Grenadines | 0.00 | 0.00 | 0.00 | 0.00 | 0.00 | 0.00 | 0.00 | 0.00 | 0.00 | 0.00 | 3.77 | 0.00 | 3.85 | 21.15 | 65.38 | 6.28 |
| Suriname | 0.00 | 0.00 | 0.00 | 0.00 | 0.00 | 0.00 | 0.00 | 0.00 | 0.00 | 0.00 | 98.11 | 100.00 | 100.00 | 98.08 | 96.15 | 32.82 |
| United States | 100.00 | 63.46 | 100.00 | 100.00 | 100.00 | 100.00 | 100.00 | 100.00 | 100.00 | 100.00 | 100.00 | 100.00 | 100.00 | 100.00 | 100.00 | 97.56 |
| Uruguay | 51.92 | 46.15 | 63.46 | 59.62 | 47.17 | 50.00 | 71.15 | 75.00 | 100.00 | 100.00 | 98.11 | 100.00 | 96.15 | 90.38 | 98.08 | 76.48 |
| Venezuela | 15.38 | 67.31 | 100.00 | 0.00 | 0.00 | 0.00 | 100.00 | 0.00 | 0.00 | 100.00 | 88.68 | 100.00 | 98.08 | 98.08 | 98.08 | 57.71 |
| **Average** | **33.49** | **36.07** | **44.69** | **51.19** | **49.12** | **70.09** | **74.73** | **71.15** | **72.21** | **75.27** | **85.17** | **83.09** | **91.45** | **92.57** | **94.89** | **68.35** |

| **Influenza A(H1) Positives** | | | | | | | | | | | | | | | | |
| --- | --- | --- | --- | --- | --- | --- | --- | --- | --- | --- | --- | --- | --- | --- | --- | --- |
| Argentina | 78.85 | 100.00 | 94.23 | 96.15 | 96.23 | 100.00 | 100.00 | 100.00 | 100.00 | 100.00 | 100.00 | 100.00 | 96.15 | 75.00 | 0.00 | 89.11 |
| Barbados | 0.00 | 0.00 | 0.00 | 0.00 | 0.00 | 0.00 | 0.00 | 0.00 | 0.00 | 0.00 | 0.00 | 0.00 | 0.00 | 0.00 | 0.00 | 0.00 |
| Belize | 0.00 | 0.00 | 0.00 | 0.00 | 0.00 | 0.00 | 0.00 | 0.00 | 0.00 | 0.00 | 0.00 | 0.00 | 98.08 | 84.62 | 100.00 | 18.85 |
| Bolivia | 0.00 | 0.00 | 0.00 | 0.00 | 0.00 | 100.00 | 100.00 | 100.00 | 100.00 | 100.00 | 90.57 | 73.08 | 100.00 | 100.00 | 100.00 | 64.24 |
| Brazil | 100.00 | 100.00 | 100.00 | 100.00 | 100.00 | 100.00 | 100.00 | 100.00 | 100.00 | 100.00 | 100.00 | 78.85 | 19.23 | 17.31 | 0.00 | 81.03 |
| Canada | 0.00 | 0.00 | 9.62 | 32.69 | 86.79 | 100.00 | 98.08 | 98.08 | 100.00 | 100.00 | 100.00 | 51.92 | 0.00 | 0.00 | 0.00 | 51.81 |
| Chile | 15.38 | 73.08 | 21.15 | 48.08 | 45.28 | 100.00 | 100.00 | 100.00 | 100.00 | 100.00 | 1.89 | 0.00 | 100.00 | 100.00 | 100.00 | 66.99 |
| Colombia | 100.00 | 0.00 | 0.00 | 0.00 | 98.11 | 100.00 | 100.00 | 100.00 | 96.15 | 100.00 | 96.23 | 0.00 | 100.00 | 0.00 | 0.00 | 59.37 |
| Costa Rica | 0.00 | 7.69 | 1.92 | 86.54 | 30.19 | 100.00 | 100.00 | 100.00 | 100.00 | 100.00 | 98.11 | 100.00 | 100.00 | 98.08 | 100.00 | 74.84 |
| Cuba | 0.00 | 0.00 | 0.00 | 0.00 | 98.11 | 100.00 | 100.00 | 98.08 | 100.00 | 100.00 | 98.11 | 100.00 | 98.08 | 100.00 | 98.08 | 72.70 |
| Dominica | 0.00 | 0.00 | 0.00 | 0.00 | 0.00 | 0.00 | 0.00 | 0.00 | 0.00 | 0.00 | 0.00 | 0.00 | 0.00 | 0.00 | 0.00 | 0.00 |
| Dominican Republic | 0.00 | 0.00 | 0.00 | 0.00 | 1.89 | 100.00 | 100.00 | 100.00 | 100.00 | 96.15 | 98.11 | 100.00 | 0.00 | 0.00 | 0.00 | 46.41 |
| Ecuador | 0.00 | 0.00 | 3.85 | 0.00 | 20.75 | 88.46 | 100.00 | 100.00 | 100.00 | 100.00 | 9.43 | 0.00 | 0.00 | 100.00 | 0.00 | 41.50 |
| El Salvador | 0.00 | 82.69 | 0.00 | 51.92 | 84.91 | 96.15 | 100.00 | 100.00 | 100.00 | 100.00 | 100.00 | 100.00 | 100.00 | 96.15 | 100.00 | 80.79 |
| Guatemala | 0.00 | 0.00 | 0.00 | 0.00 | 33.96 | 98.08 | 100.00 | 96.15 | 100.00 | 94.23 | 98.11 | 100.00 | 90.38 | 0.00 | 0.00 | 54.06 |
| Haiti | 0.00 | 0.00 | 0.00 | 0.00 | 0.00 | 0.00 | 0.00 | 0.00 | 0.00 | 0.00 | 24.53 | 0.00 | 100.00 | 100.00 | 78.85 | 20.22 |
| Honduras | 0.00 | 0.00 | 100.00 | 98.08 | 98.11 | 100.00 | 100.00 | 96.15 | 98.08 | 92.31 | 98.11 | 100.00 | 0.00 | 100.00 | 0.00 | 72.06 |
| Jamaica | 0.00 | 0.00 | 0.00 | 100.00 | 98.11 | 100.00 | 98.08 | 100.00 | 100.00 | 100.00 | 100.00 | 96.15 | 100.00 | 98.08 | 100.00 | 79.36 |
| Mexico | 100.00 | 100.00 | 100.00 | 100.00 | 100.00 | 100.00 | 100.00 | 100.00 | 100.00 | 100.00 | 98.11 | 100.00 | 98.08 | 100.00 | 100.00 | 99.75 |
| Nicaragua | 0.00 | 0.00 | 0.00 | 0.00 | 0.00 | 100.00 | 100.00 | 100.00 | 100.00 | 100.00 | 1.89 | 0.00 | 0.00 | 0.00 | 0.00 | 33.46 |
| Panama | 0.00 | 0.00 | 0.00 | 100.00 | 20.75 | 100.00 | 100.00 | 100.00 | 100.00 | 100.00 | 100.00 | 100.00 | 100.00 | 100.00 | 100.00 | 74.72 |
| Paraguay | 67.31 | 30.77 | 0.00 | 98.08 | 100.00 | 100.00 | 100.00 | 100.00 | 100.00 | 100.00 | 98.11 | 100.00 | 100.00 | 100.00 | 100.00 | 86.28 |
| Peru | 100.00 | 30.77 | 11.54 | 5.77 | 5.66 | 100.00 | 100.00 | 100.00 | 100.00 | 100.00 | 100.00 | 98.08 | 100.00 | 82.69 | 100.00 | 75.63 |
| St. Lucia | 0.00 | 0.00 | 0.00 | 0.00 | 0.00 | 0.00 | 0.00 | 0.00 | 0.00 | 0.00 | 0.00 | 0.00 | 0.00 | 0.00 | 25.00 | 1.67 |
| St. Vincent & the Grenadines | 0.00 | 0.00 | 0.00 | 0.00 | 0.00 | 0.00 | 0.00 | 0.00 | 0.00 | 0.00 | 0.00 | 0.00 | 0.00 | 0.00 | 0.00 | 0.00 |
| Suriname | 0.00 | 0.00 | 0.00 | 0.00 | 0.00 | 0.00 | 0.00 | 0.00 | 0.00 | 0.00 | 98.11 | 100.00 | 53.85 | 98.08 | 96.15 | 29.75 |
| United States | 100.00 | 63.46 | 100.00 | 100.00 | 100.00 | 100.00 | 100.00 | 100.00 | 100.00 | 100.00 | 100.00 | 100.00 | 100.00 | 100.00 | 100.00 | 97.56 |
| Uruguay | 51.92 | 21.15 | 63.46 | 59.62 | 18.87 | 50.00 | 71.15 | 75.00 | 100.00 | 100.00 | 98.11 | 0.00 | 96.15 | 90.38 | 98.08 | 66.26 |
| Venezuela | 0.00 | 32.69 | 98.08 | 0.00 | 0.00 | 0.00 | 100.00 | 0.00 | 0.00 | 100.00 | 88.68 | 100.00 | 98.08 | 98.08 | 98.08 | 54.25 |
| **Average** | **24.60** | **22.15** | **24.27** | **37.14** | **42.68** | **70.09** | **74.73** | **71.15** | **72.21** | **75.27** | **68.84** | **58.55** | **63.73** | **63.40** | **54.97** | **54.92** |

| **Influenza A(H1N1)pdm09 Positives** | | | | | | | | | | | | | | | | |
| --- | --- | --- | --- | --- | --- | --- | --- | --- | --- | --- | --- | --- | --- | --- | --- | --- |
| Argentina | - | - | - | 0.00 | 73.58 | 100.00 | 100.00 | 100.00 | 100.00 | 100.00 | 100.00 | 100.00 | 100.00 | 100.00 | 100.00 | 89.47 |
| Barbados | - | - | - | 0.00 | 0.00 | 0.00 | 0.00 | 0.00 | 0.00 | 0.00 | 15.09 | 9.62 | 0.00 | 7.69 | 19.23 | 4.30 |
| Belize | - | - | - | 0.00 | 0.00 | 0.00 | 0.00 | 0.00 | 0.00 | 0.00 | 0.00 | 0.00 | 98.08 | 84.62 | 100.00 | 23.56 |
| Bolivia | - | - | - | 0.00 | 0.00 | 100.00 | 100.00 | 100.00 | 100.00 | 100.00 | 96.23 | 75.00 | 100.00 | 100.00 | 100.00 | 80.94 |
| Brazil | - | - | - | 0.00 | 67.92 | 100.00 | 100.00 | 100.00 | 100.00 | 100.00 | 100.00 | 98.08 | 100.00 | 90.38 | 96.15 | 87.71 |
| Canada | - | - | - | 0.00 | 86.79 | 100.00 | 98.08 | 98.08 | 100.00 | 100.00 | 100.00 | 100.00 | 100.00 | 100.00 | 100.00 | 90.25 |
| Chile | - | - | - | 0.00 | 69.81 | 100.00 | 100.00 | 100.00 | 100.00 | 100.00 | 56.60 | 82.69 | 100.00 | 100.00 | 100.00 | 84.09 |
| Colombia | - | - | - | 0.00 | 98.11 | 100.00 | 100.00 | 100.00 | 96.15 | 100.00 | 96.23 | 100.00 | 100.00 | 100.00 | 100.00 | 90.87 |
| Costa Rica | - | - | - | 0.00 | 1.89 | 100.00 | 100.00 | 100.00 | 100.00 | 100.00 | 98.11 | 100.00 | 100.00 | 98.08 | 100.00 | 83.17 |
| Cuba | - | - | - | 0.00 | 98.11 | 100.00 | 100.00 | 98.08 | 100.00 | 100.00 | 98.11 | 100.00 | 98.08 | 100.00 | 98.08 | 90.87 |
| Dominica | - | - | - | 0.00 | 0.00 | 0.00 | 0.00 | 0.00 | 0.00 | 0.00 | 9.43 | 0.00 | 0.00 | 9.62 | 3.85 | 1.91 |
| Dominican Republic | - | - | - | 0.00 | 0.00 | 100.00 | 100.00 | 100.00 | 100.00 | 96.15 | 98.11 | 100.00 | 1.92 | 69.23 | 19.23 | 65.39 |
| Ecuador | - | - | - | 0.00 | 41.51 | 88.46 | 100.00 | 100.00 | 100.00 | 100.00 | 7.55 | 65.38 | 19.23 | 100.00 | 50.00 | 64.34 |
| El Salvador | - | - | - | 0.00 | 84.91 | 96.15 | 100.00 | 100.00 | 100.00 | 100.00 | 100.00 | 100.00 | 100.00 | 96.15 | 100.00 | 89.77 |
| Guatemala | - | - | - | 0.00 | 33.96 | 98.08 | 100.00 | 96.15 | 100.00 | 94.23 | 98.11 | 100.00 | 92.31 | 57.69 | 76.92 | 78.96 |
| Haiti | - | - | - | 0.00 | 0.00 | 0.00 | 0.00 | 0.00 | 0.00 | 0.00 | 24.53 | 26.92 | 100.00 | 100.00 | 78.85 | 27.52 |
| Honduras | - | - | - | 0.00 | 98.11 | 100.00 | 100.00 | 96.15 | 98.08 | 92.31 | 98.11 | 100.00 | 98.08 | 100.00 | 98.08 | 89.91 |
| Jamaica | - | - | - | 100.00 | 98.11 | 100.00 | 98.08 | 100.00 | 100.00 | 100.00 | 100.00 | 96.15 | 100.00 | 98.08 | 100.00 | 99.20 |
| Mexico | - | - | - | 1.92 | 100.00 | 100.00 | 100.00 | 100.00 | 100.00 | 100.00 | 98.11 | 100.00 | 100.00 | 100.00 | 100.00 | 91.67 |
| Nicaragua | - | - | - | 0.00 | 0.00 | 100.00 | 100.00 | 100.00 | 100.00 | 100.00 | 22.64 | 11.54 | 3.85 | 44.23 | 51.92 | 52.85 |
| Panama | - | - | - | 0.00 | 1.89 | 100.00 | 100.00 | 100.00 | 100.00 | 100.00 | 100.00 | 100.00 | 100.00 | 100.00 | 100.00 | 83.49 |
| Paraguay | - | - | - | 7.69 | 100.00 | 100.00 | 100.00 | 100.00 | 100.00 | 100.00 | 98.11 | 100.00 | 100.00 | 100.00 | 100.00 | 92.15 |
| Peru | - | - | - | 0.00 | 0.00 | 100.00 | 100.00 | 100.00 | 100.00 | 100.00 | 100.00 | 98.08 | 100.00 | 82.69 | 100.00 | 81.73 |
| St. Lucia | - | - | - | 0.00 | 0.00 | 0.00 | 0.00 | 0.00 | 0.00 | 0.00 | 0.00 | 0.00 | 0.00 | 7.69 | 25.00 | 2.72 |
| St. Vincent & the Grenadines | - | - | - | 0.00 | 0.00 | 0.00 | 0.00 | 0.00 | 0.00 | 0.00 | 0.00 | 0.00 | 0.00 | 13.46 | 1.92 | 1.28 |
| Suriname | - | - | - | 0.00 | 0.00 | 0.00 | 0.00 | 0.00 | 0.00 | 0.00 | 98.11 | 100.00 | 53.85 | 98.08 | 96.15 | 37.18 |
| United States | - | - | - | 0.00 | 73.58 | 100.00 | 100.00 | 100.00 | 100.00 | 100.00 | 100.00 | 100.00 | 100.00 | 100.00 | 100.00 | 89.47 |
| Uruguay | - | - | - | 0.00 | 32.08 | 50.00 | 71.15 | 75.00 | 100.00 | 100.00 | 98.11 | 21.15 | 96.15 | 90.38 | 96.15 | 69.18 |
| Venezuela | - | - | - | 0.00 | 0.00 | 0.00 | 100.00 | 0.00 | 0.00 | 100.00 | 88.68 | 100.00 | 98.08 | 98.08 | 98.08 | 56.91 |
| **Average** | - | - | - | **3.78** | **40.01** | **70.09** | **74.73** | **71.15** | **72.21** | **75.27** | **72.41** | **71.88** | **74.47** | **80.90** | **79.64** | **65.55** |

| **Influenza A(H5) Positives** | | | | | | | | | | | | | | | | |
| --- | --- | --- | --- | --- | --- | --- | --- | --- | --- | --- | --- | --- | --- | --- | --- | --- |
| Argentina | - | - | - | 0.00 | 73.58 | 100.00 | 100.00 | 100.00 | 100.00 | 96.15 | 94.34 | 53.85 | - | - | - | 79.77 |
| Barbados | - | - | - | 0.00 | 0.00 | 0.00 | 0.00 | 0.00 | 0.00 | 0.00 | 0.00 | 0.00 | - | - | - | 0.00 |
| Belize | - | - | - | 0.00 | 0.00 | 0.00 | 0.00 | 0.00 | 0.00 | 0.00 | 0.00 | 0.00 | - | - | - | 0.00 |
| Bolivia | - | - | - | 0.00 | 0.00 | 0.00 | 0.00 | 0.00 | 0.00 | 0.00 | 0.00 | 0.00 | - | - | - | 0.00 |
| Brazil | - | - | - | 0.00 | 67.92 | 100.00 | 100.00 | 100.00 | 100.00 | 100.00 | 100.00 | 78.85 | - | - | - | 82.97 |
| Canada | - | - | - | 0.00 | 1.89 | 84.62 | 98.08 | 98.08 | 100.00 | 100.00 | 100.00 | 51.92 | - | - | - | 70.51 |
| Chile | - | - | - | 0.00 | 33.96 | 100.00 | 100.00 | 100.00 | 55.77 | 0.00 | 0.00 | 0.00 | - | - | - | 43.30 |
| Colombia | - | - | - | 0.00 | 98.11 | 67.31 | 25.00 | 0.00 | 0.00 | 0.00 | 0.00 | 0.00 | - | - | - | 21.16 |
| Costa Rica | - | - | - | 0.00 | 0.00 | 23.08 | 42.31 | 19.23 | 0.00 | 0.00 | 0.00 | 0.00 | - | - | - | 9.40 |
| Cuba | - | - | - | 0.00 | 98.11 | 30.77 | 0.00 | 0.00 | 0.00 | 0.00 | 0.00 | 0.00 | - | - | - | 14.32 |
| Dominica | - | - | - | 0.00 | 0.00 | 0.00 | 0.00 | 0.00 | 0.00 | 0.00 | 0.00 | 0.00 | - | - | - | 0.00 |
| Dominican Republic | - | - | - | 0.00 | 0.00 | 0.00 | 0.00 | 0.00 | 0.00 | 0.00 | 0.00 | 0.00 | - | - | - | 0.00 |
| Ecuador | - | - | - | 0.00 | 3.77 | 0.00 | 0.00 | 0.00 | 0.00 | 0.00 | 0.00 | 0.00 | - | - | - | 0.42 |
| El Salvador | - | - | - | 0.00 | 0.00 | 86.54 | 96.15 | 76.92 | 65.38 | 86.54 | 49.06 | 0.00 | - | - | - | 51.18 |
| Guatemala | - | - | - | 0.00 | 33.96 | 92.31 | 15.38 | 7.69 | 65.38 | 0.00 | 0.00 | 0.00 | - | - | - | 23.86 |
| Haiti | - | - | - | 0.00 | 0.00 | 0.00 | 0.00 | 0.00 | 0.00 | 0.00 | 0.00 | 0.00 | - | - | - | 0.00 |
| Honduras | - | - | - | 0.00 | 98.11 | 100.00 | 100.00 | 28.85 | 0.00 | 0.00 | 0.00 | 0.00 | - | - | - | 36.33 |
| Jamaica | - | - | - | 100.00 | 98.11 | 100.00 | 69.23 | 1.92 | 1.92 | 0.00 | 0.00 | 0.00 | - | - | - | 41.24 |
| Mexico | - | - | - | 1.92 | 100.00 | 100.00 | 100.00 | 100.00 | 94.23 | 96.15 | 43.40 | 0.00 | - | - | - | 70.63 |
| Nicaragua | - | - | - | 0.00 | 0.00 | 0.00 | 0.00 | 0.00 | 0.00 | 0.00 | 0.00 | 0.00 | - | - | - | 0.00 |
| Panama | - | - | - | 0.00 | 0.00 | 100.00 | 100.00 | 98.08 | 90.38 | 19.23 | 0.00 | 0.00 | - | - | - | 45.30 |
| Paraguay | - | - | - | 7.69 | 100.00 | 26.92 | 0.00 | 0.00 | 0.00 | 0.00 | 0.00 | 0.00 | - | - | - | 14.96 |
| Peru | - | - | - | 0.00 | 0.00 | 100.00 | 36.54 | 94.23 | 0.00 | 0.00 | 0.00 | 0.00 | - | - | - | 25.64 |
| St. Lucia | - | - | - | 0.00 | 0.00 | 0.00 | 0.00 | 0.00 | 0.00 | 0.00 | 0.00 | 0.00 | - | - | - | 0.00 |
| St. Vincent & the Grenadines | - | - | - | 0.00 | 0.00 | 0.00 | 0.00 | 0.00 | 0.00 | 0.00 | 0.00 | 0.00 | - | - | - | 0.00 |
| Suriname | - | - | - | 0.00 | 0.00 | 0.00 | 0.00 | 0.00 | 0.00 | 0.00 | 0.00 | 0.00 | - | - | - | 0.00 |
| United States | - | - | - | 0.00 | 33.96 | 100.00 | 100.00 | 100.00 | 100.00 | 100.00 | 75.47 | 0.00 | - | - | - | 67.71 |
| Uruguay | - | - | - | 0.00 | 0.00 | 0.00 | 0.00 | 0.00 | 0.00 | 0.00 | 0.00 | 0.00 | - | - | - | 0.00 |
| Venezuela | - | - | - | 0.00 | 0.00 | 0.00 | 0.00 | 0.00 | 0.00 | 100.00 | 67.92 | 0.00 | - | - | - | 18.66 |
| **Average** | - | - | - | **3.78** | **29.02** | **45.23** | **37.33** | **31.90** | **26.66** | **24.07** | **18.28** | **6.37** | - | - | - | **24.74** |

| **Influenza A(H3) Positives** | | | | | | | | | | | | | | | | |
| --- | --- | --- | --- | --- | --- | --- | --- | --- | --- | --- | --- | --- | --- | --- | --- | --- |
| Argentina | 88.46 | 100.00 | 94.23 | 96.15 | 96.23 | 100.00 | 100.00 | 100.00 | 100.00 | 100.00 | 100.00 | 100.00 | 100.00 | 100.00 | 100.00 | 98.34 |
| Barbados | 0.00 | 0.00 | 0.00 | 0.00 | 0.00 | 0.00 | 0.00 | 0.00 | 0.00 | 0.00 | 3.77 | 7.69 | 5.77 | 3.85 | 19.23 | 2.69 |
| Belize | 0.00 | 0.00 | 0.00 | 0.00 | 0.00 | 0.00 | 0.00 | 0.00 | 0.00 | 0.00 | 0.00 | 0.00 | 98.08 | 84.62 | 100.00 | 18.85 |
| Bolivia | 0.00 | 0.00 | 0.00 | 0.00 | 0.00 | 100.00 | 100.00 | 100.00 | 100.00 | 100.00 | 92.45 | 73.08 | 100.00 | 100.00 | 100.00 | 64.37 |
| Brazil | 100.00 | 100.00 | 100.00 | 100.00 | 100.00 | 100.00 | 100.00 | 100.00 | 100.00 | 100.00 | 100.00 | 92.31 | 100.00 | 98.08 | 90.38 | 98.72 |
| Canada | 0.00 | 0.00 | 5.77 | 26.92 | 86.79 | 100.00 | 98.08 | 98.08 | 100.00 | 100.00 | 100.00 | 100.00 | 100.00 | 100.00 | 100.00 | 74.38 |
| Chile | 42.31 | 40.38 | 34.62 | 0.00 | 50.94 | 100.00 | 100.00 | 100.00 | 100.00 | 100.00 | 71.70 | 71.15 | 100.00 | 100.00 | 100.00 | 74.07 |
| Colombia | 100.00 | 0.00 | 0.00 | 0.00 | 98.11 | 100.00 | 100.00 | 100.00 | 96.15 | 100.00 | 96.23 | 100.00 | 100.00 | 100.00 | 100.00 | 79.37 |
| Costa Rica | 5.77 | 15.38 | 3.85 | 86.54 | 30.19 | 100.00 | 100.00 | 100.00 | 100.00 | 100.00 | 98.11 | 100.00 | 100.00 | 98.08 | 100.00 | 75.86 |
| Cuba | 0.00 | 0.00 | 0.00 | 0.00 | 98.11 | 100.00 | 100.00 | 98.08 | 100.00 | 100.00 | 98.11 | 100.00 | 98.08 | 100.00 | 98.08 | 72.70 |
| Dominica | 0.00 | 0.00 | 0.00 | 0.00 | 0.00 | 0.00 | 0.00 | 0.00 | 0.00 | 0.00 | 5.66 | 0.00 | 0.00 | 0.00 | 1.92 | 0.51 |
| Dominican Republic | 1.92 | 0.00 | 0.00 | 0.00 | 1.89 | 100.00 | 100.00 | 100.00 | 100.00 | 96.15 | 98.11 | 100.00 | 15.38 | 0.00 | 42.31 | 50.38 |
| Ecuador | 0.00 | 0.00 | 0.00 | 0.00 | 30.19 | 88.46 | 100.00 | 100.00 | 100.00 | 100.00 | 66.04 | 57.69 | 61.54 | 100.00 | 32.69 | 55.77 |
| El Salvador | 0.00 | 82.69 | 0.00 | 51.92 | 84.91 | 96.15 | 100.00 | 100.00 | 100.00 | 100.00 | 100.00 | 100.00 | 100.00 | 96.15 | 100.00 | 80.79 |
| Guatemala | 0.00 | 0.00 | 0.00 | 0.00 | 33.96 | 98.08 | 100.00 | 96.15 | 100.00 | 94.23 | 98.11 | 100.00 | 92.31 | 55.77 | 53.85 | 61.50 |
| Haiti | 0.00 | 0.00 | 0.00 | 0.00 | 0.00 | 0.00 | 0.00 | 0.00 | 0.00 | 0.00 | 24.53 | 26.92 | 100.00 | 100.00 | 78.85 | 22.02 |
| Honduras | 0.00 | 0.00 | 100.00 | 98.08 | 98.11 | 100.00 | 100.00 | 96.15 | 98.08 | 92.31 | 98.11 | 100.00 | 98.08 | 100.00 | 98.08 | 85.13 |
| Jamaica | 0.00 | 0.00 | 0.00 | 100.00 | 98.11 | 100.00 | 98.08 | 100.00 | 100.00 | 100.00 | 100.00 | 96.15 | 100.00 | 98.08 | 100.00 | 79.36 |
| Mexico | 100.00 | 100.00 | 100.00 | 98.08 | 100.00 | 100.00 | 100.00 | 100.00 | 100.00 | 100.00 | 98.11 | 100.00 | 100.00 | 100.00 | 100.00 | 99.75 |
| Nicaragua | 0.00 | 0.00 | 0.00 | 0.00 | 0.00 | 100.00 | 100.00 | 100.00 | 100.00 | 100.00 | 20.75 | 25.00 | 48.08 | 5.77 | 53.85 | 43.56 |
| Panama | 0.00 | 0.00 | 0.00 | 98.08 | 20.75 | 100.00 | 100.00 | 100.00 | 100.00 | 100.00 | 100.00 | 100.00 | 100.00 | 100.00 | 100.00 | 74.59 |
| Paraguay | 80.77 | 30.77 | 0.00 | 98.08 | 100.00 | 100.00 | 100.00 | 100.00 | 100.00 | 100.00 | 98.11 | 100.00 | 100.00 | 100.00 | 100.00 | 87.18 |
| Peru | 100.00 | 32.69 | 11.54 | 1.92 | 0.00 | 100.00 | 100.00 | 100.00 | 100.00 | 100.00 | 100.00 | 98.08 | 100.00 | 82.69 | 100.00 | 75.13 |
| St. Lucia | 0.00 | 0.00 | 0.00 | 0.00 | 0.00 | 0.00 | 0.00 | 0.00 | 0.00 | 0.00 | 0.00 | 0.00 | 0.00 | 5.77 | 25.00 | 2.05 |
| St. Vincent & the Grenadines | 0.00 | 0.00 | 0.00 | 0.00 | 0.00 | 0.00 | 0.00 | 0.00 | 0.00 | 0.00 | 3.77 | 0.00 | 0.00 | 1.92 | 3.85 | 0.64 |
| Suriname | 0.00 | 0.00 | 0.00 | 0.00 | 0.00 | 0.00 | 0.00 | 0.00 | 0.00 | 0.00 | 98.11 | 100.00 | 63.46 | 98.08 | 96.15 | 30.39 |
| United States | 100.00 | 63.46 | 100.00 | 100.00 | 100.00 | 100.00 | 100.00 | 100.00 | 100.00 | 100.00 | 100.00 | 100.00 | 100.00 | 100.00 | 100.00 | 97.56 |
| Uruguay | 51.92 | 21.15 | 63.46 | 59.62 | 16.98 | 50.00 | 71.15 | 75.00 | 100.00 | 100.00 | 98.11 | 0.00 | 96.15 | 90.38 | 98.08 | 66.13 |
| Venezuela | 15.38 | 13.46 | 100.00 | 0.00 | 0.00 | 0.00 | 100.00 | 0.00 | 0.00 | 100.00 | 88.68 | 100.00 | 98.08 | 98.08 | 98.08 | 54.12 |
| **Average** | **27.12** | **20.69** | **24.60** | **35.01** | **42.94** | **70.09** | **74.73** | **71.15** | **72.21** | **75.27** | **74.37** | **70.62** | **78.45** | **76.46** | **78.98** | **59.51** |

| **Influenza A(Unsubtyped) Positives** | | | | | | | | | | | | | | | | |
| --- | --- | --- | --- | --- | --- | --- | --- | --- | --- | --- | --- | --- | --- | --- | --- | --- |
| Argentina | 88.46 | 100.00 | 94.23 | 96.15 | 96.23 | 100.00 | 100.00 | 100.00 | 100.00 | 100.00 | 100.00 | 100.00 | 100.00 | 100.00 | 100.00 | 98.34 |
| Barbados | 0.00 | 0.00 | 0.00 | 0.00 | 0.00 | 0.00 | 0.00 | 0.00 | 0.00 | 0.00 | 3.77 | 0.00 | 0.00 | 0.00 | 0.00 | 0.25 |
| Belize | 0.00 | 0.00 | 0.00 | 0.00 | 0.00 | 0.00 | 0.00 | 0.00 | 0.00 | 0.00 | 0.00 | 0.00 | 98.08 | 84.62 | 100.00 | 18.85 |
| Bolivia | 0.00 | 0.00 | 0.00 | 0.00 | 0.00 | 0.00 | 100.00 | 100.00 | 100.00 | 100.00 | 90.57 | 73.08 | 100.00 | 100.00 | 100.00 | 57.58 |
| Brazil | 100.00 | 100.00 | 100.00 | 100.00 | 100.00 | 100.00 | 100.00 | 100.00 | 100.00 | 100.00 | 100.00 | 78.85 | 100.00 | 17.31 | 0.00 | 86.41 |
| Canada | 0.00 | 34.62 | 94.23 | 100.00 | 86.79 | 100.00 | 98.08 | 98.08 | 100.00 | 100.00 | 100.00 | 100.00 | 100.00 | 100.00 | 100.00 | 87.45 |
| Chile | 63.46 | 71.15 | 76.92 | 86.54 | 52.83 | 100.00 | 100.00 | 100.00 | 100.00 | 100.00 | 35.85 | 63.46 | 100.00 | 100.00 | 100.00 | 83.35 |
| Colombia | 100.00 | 94.23 | 100.00 | 80.77 | 98.11 | 100.00 | 100.00 | 100.00 | 96.15 | 100.00 | 96.23 | 0.00 | 100.00 | 0.00 | 0.00 | 77.70 |
| Costa Rica | 0.00 | 0.00 | 32.69 | 98.08 | 41.51 | 100.00 | 100.00 | 100.00 | 100.00 | 100.00 | 98.11 | 100.00 | 100.00 | 98.08 | 100.00 | 77.90 |
| Cuba | 0.00 | 0.00 | 0.00 | 0.00 | 98.11 | 100.00 | 100.00 | 98.08 | 100.00 | 100.00 | 98.11 | 100.00 | 98.08 | 100.00 | 98.08 | 72.70 |
| Dominica | 0.00 | 0.00 | 0.00 | 0.00 | 0.00 | 0.00 | 0.00 | 0.00 | 0.00 | 0.00 | 0.00 | 0.00 | 0.00 | 0.00 | 0.00 | 0.00 |
| Dominican Republic | 98.08 | 100.00 | 100.00 | 100.00 | 16.98 | 100.00 | 100.00 | 100.00 | 100.00 | 96.15 | 98.11 | 100.00 | 17.31 | 0.00 | 0.00 | 75.11 |
| Ecuador | 0.00 | 0.00 | 3.85 | 0.00 | 22.64 | 0.00 | 100.00 | 100.00 | 100.00 | 100.00 | 0.00 | 0.00 | 0.00 | 100.00 | 0.00 | 35.10 |
| El Salvador | 0.00 | 82.69 | 0.00 | 53.85 | 83.02 | 86.54 | 100.00 | 100.00 | 100.00 | 100.00 | 100.00 | 100.00 | 100.00 | 96.15 | 100.00 | 80.15 |
| Guatemala | 0.00 | 0.00 | 0.00 | 0.00 | 33.96 | 92.31 | 100.00 | 96.15 | 100.00 | 94.23 | 98.11 | 100.00 | 92.31 | 30.77 | 38.46 | 58.42 |
| Haiti | 0.00 | 0.00 | 0.00 | 0.00 | 0.00 | 0.00 | 0.00 | 0.00 | 0.00 | 0.00 | 24.53 | 0.00 | 100.00 | 100.00 | 78.85 | 20.22 |
| Honduras | 0.00 | 0.00 | 100.00 | 98.08 | 98.11 | 100.00 | 100.00 | 96.15 | 98.08 | 92.31 | 98.11 | 100.00 | 98.08 | 100.00 | 98.08 | 85.13 |
| Jamaica | 0.00 | 0.00 | 0.00 | 100.00 | 98.11 | 100.00 | 98.08 | 100.00 | 100.00 | 100.00 | 100.00 | 96.15 | 100.00 | 98.08 | 100.00 | 79.36 |
| Mexico | 100.00 | 98.08 | 100.00 | 96.15 | 100.00 | 100.00 | 100.00 | 100.00 | 100.00 | 100.00 | 98.11 | 100.00 | 100.00 | 100.00 | 100.00 | 99.49 |
| Nicaragua | 0.00 | 0.00 | 0.00 | 0.00 | 0.00 | 100.00 | 100.00 | 100.00 | 100.00 | 100.00 | 1.89 | 0.00 | 0.00 | 19.23 | 34.62 | 37.05 |
| Panama | 0.00 | 0.00 | 46.15 | 98.08 | 22.64 | 100.00 | 100.00 | 100.00 | 100.00 | 100.00 | 100.00 | 100.00 | 100.00 | 100.00 | 100.00 | 77.79 |
| Paraguay | 88.46 | 30.77 | 0.00 | 98.08 | 100.00 | 26.92 | 100.00 | 100.00 | 100.00 | 100.00 | 98.11 | 100.00 | 100.00 | 100.00 | 100.00 | 82.82 |
| Peru | 100.00 | 98.08 | 92.31 | 98.08 | 39.62 | 100.00 | 100.00 | 100.00 | 100.00 | 100.00 | 100.00 | 98.08 | 100.00 | 82.69 | 100.00 | 93.92 |
| St. Lucia | 0.00 | 0.00 | 0.00 | 0.00 | 0.00 | 0.00 | 0.00 | 0.00 | 0.00 | 0.00 | 0.00 | 0.00 | 0.00 | 0.00 | 25.00 | 1.67 |
| St. Vincent & the Grenadines | 0.00 | 0.00 | 0.00 | 0.00 | 0.00 | 0.00 | 0.00 | 0.00 | 0.00 | 0.00 | 0.00 | 0.00 | 0.00 | 0.00 | 0.00 | 0.00 |
| Suriname | 0.00 | 0.00 | 0.00 | 0.00 | 0.00 | 0.00 | 0.00 | 0.00 | 0.00 | 0.00 | 98.11 | 100.00 | 53.85 | 98.08 | 96.15 | 29.75 |
| United States | 100.00 | 63.46 | 100.00 | 100.00 | 100.00 | 100.00 | 100.00 | 100.00 | 100.00 | 100.00 | 100.00 | 100.00 | 100.00 | 100.00 | 100.00 | 97.56 |
| Uruguay | 50.00 | 46.15 | 63.46 | 59.62 | 45.28 | 0.00 | 71.15 | 75.00 | 100.00 | 100.00 | 98.11 | 0.00 | 96.15 | 90.38 | 98.08 | 66.23 |
| Venezuela | 1.92 | 0.00 | 100.00 | 0.00 | 0.00 | 0.00 | 100.00 | 0.00 | 0.00 | 100.00 | 88.68 | 100.00 | 98.08 | 98.08 | 98.08 | 52.32 |
| **Average** | **30.70** | **31.70** | **41.51** | **50.46** | **46.00** | **58.82** | **74.73** | **71.15** | **72.21** | **75.27** | **69.81** | **62.40** | **74.20** | **69.43** | **67.77** | **59.75** |

| **Influenza B(Yamataga) Positives** | | | | | | | | | | | | | | | | |
| --- | --- | --- | --- | --- | --- | --- | --- | --- | --- | --- | --- | --- | --- | --- | --- | --- |
| Argentina | - | - | 0.00 | 96.15 | 96.23 | 100.00 | 100.00 | 100.00 | 100.00 | 100.00 | 100.00 | 100.00 | 100.00 | 100.00 | 100.00 | 91.72 |
| Barbados | - | - | 0.00 | 0.00 | 0.00 | 0.00 | 0.00 | 0.00 | 0.00 | 0.00 | 0.00 | 0.00 | 0.00 | 7.69 | 0.00 | 0.59 |
| Belize | - | - | 0.00 | 0.00 | 0.00 | 0.00 | 0.00 | 0.00 | 0.00 | 0.00 | 0.00 | 0.00 | 98.08 | 84.62 | 100.00 | 21.75 |
| Bolivia | - | - | 0.00 | 0.00 | 0.00 | 0.00 | 0.00 | 0.00 | 100.00 | 100.00 | 90.57 | 73.08 | 100.00 | 100.00 | 100.00 | 51.05 |
| Brazil | - | - | 9.62 | 100.00 | 100.00 | 100.00 | 100.00 | 100.00 | 100.00 | 100.00 | 100.00 | 78.85 | 92.31 | 90.38 | 28.85 | 84.62 |
| Canada | - | - | 0.00 | 0.00 | 1.89 | 84.62 | 98.08 | 98.08 | 100.00 | 100.00 | 100.00 | 51.92 | 0.00 | 0.00 | 0.00 | 48.81 |
| Chile | - | - | 0.00 | 0.00 | 33.96 | 100.00 | 100.00 | 100.00 | 100.00 | 100.00 | 75.47 | 76.92 | 100.00 | 100.00 | 100.00 | 75.87 |
| Colombia | - | - | 0.00 | 0.00 | 98.11 | 67.31 | 25.00 | 0.00 | 53.85 | 100.00 | 96.23 | 0.00 | 0.00 | 75.00 | 100.00 | 47.35 |
| Costa Rica | - | - | 0.00 | 88.46 | 30.19 | 23.08 | 42.31 | 19.23 | 46.15 | 100.00 | 0.00 | 0.00 | 0.00 | 0.00 | 100.00 | 34.57 |
| Cuba | - | - | 0.00 | 0.00 | 98.11 | 30.77 | 0.00 | 0.00 | 59.62 | 100.00 | 98.11 | 0.00 | 98.08 | 100.00 | 98.08 | 52.52 |
| Dominica | - | - | 0.00 | 0.00 | 0.00 | 0.00 | 0.00 | 0.00 | 0.00 | 0.00 | 3.77 | 0.00 | 0.00 | 0.00 | 0.00 | 0.29 |
| Dominican Republic | - | - | 0.00 | 0.00 | 0.00 | 0.00 | 0.00 | 0.00 | 50.00 | 96.15 | 0.00 | 100.00 | 0.00 | 21.15 | 19.23 | 22.04 |
| Ecuador | - | - | 0.00 | 0.00 | 3.77 | 0.00 | 0.00 | 0.00 | 50.00 | 100.00 | 5.66 | 0.00 | 0.00 | 100.00 | 0.00 | 19.96 |
| El Salvador | - | - | 0.00 | 50.00 | 84.91 | 86.54 | 96.15 | 76.92 | 80.77 | 100.00 | 100.00 | 100.00 | 100.00 | 96.15 | 100.00 | 82.42 |
| Guatemala | - | - | 0.00 | 0.00 | 33.96 | 92.31 | 15.38 | 7.69 | 100.00 | 94.23 | 98.11 | 100.00 | 92.31 | 1.92 | 21.15 | 50.54 |
| Haiti | - | - | 0.00 | 0.00 | 0.00 | 0.00 | 0.00 | 0.00 | 0.00 | 0.00 | 24.53 | 26.92 | 100.00 | 100.00 | 78.85 | 25.41 |
| Honduras | - | - | 0.00 | 32.69 | 98.11 | 100.00 | 100.00 | 28.85 | 88.46 | 92.31 | 0.00 | 100.00 | 98.08 | 100.00 | 98.08 | 72.04 |
| Jamaica | - | - | 0.00 | 100.00 | 98.11 | 100.00 | 69.23 | 1.92 | 48.08 | 100.00 | 100.00 | 61.54 | 100.00 | 98.08 | 100.00 | 75.15 |
| Mexico | - | - | 0.00 | 17.31 | 100.00 | 100.00 | 100.00 | 100.00 | 100.00 | 100.00 | 0.00 | 0.00 | 100.00 | 100.00 | 100.00 | 70.56 |
| Nicaragua | - | - | 0.00 | 0.00 | 0.00 | 0.00 | 0.00 | 0.00 | 48.08 | 100.00 | 1.89 | 0.00 | 0.00 | 0.00 | 0.00 | 11.54 |
| Panama | - | - | 0.00 | 98.08 | 20.75 | 100.00 | 100.00 | 98.08 | 100.00 | 100.00 | 100.00 | 100.00 | 100.00 | 100.00 | 100.00 | 85.92 |
| Paraguay | - | - | 0.00 | 96.15 | 100.00 | 26.92 | 0.00 | 0.00 | 51.92 | 100.00 | 98.11 | 100.00 | 100.00 | 100.00 | 100.00 | 67.16 |
| Peru | - | - | 0.00 | 0.00 | 0.00 | 100.00 | 36.54 | 94.23 | 42.31 | 100.00 | 100.00 | 98.08 | 100.00 | 82.69 | 100.00 | 65.68 |
| St. Lucia | - | - | 0.00 | 0.00 | 0.00 | 0.00 | 0.00 | 0.00 | 0.00 | 0.00 | 0.00 | 0.00 | 0.00 | 1.92 | 25.00 | 2.07 |
| St. Vincent & the Grenadines | - | - | 0.00 | 0.00 | 0.00 | 0.00 | 0.00 | 0.00 | 0.00 | 0.00 | 0.00 | 0.00 | 0.00 | 0.00 | 0.00 | 0.00 |
| Suriname | - | - | 0.00 | 0.00 | 0.00 | 0.00 | 0.00 | 0.00 | 0.00 | 0.00 | 98.11 | 100.00 | 53.85 | 98.08 | 96.15 | 34.32 |
| United States | - | - | 0.00 | 0.00 | 33.96 | 100.00 | 100.00 | 100.00 | 100.00 | 100.00 | 100.00 | 100.00 | 100.00 | 100.00 | 100.00 | 79.54 |
| Uruguay | - | - | 0.00 | 17.31 | 13.21 | 0.00 | 0.00 | 0.00 | 57.69 | 100.00 | 0.00 | 1.92 | 96.15 | 90.38 | 98.08 | 36.52 |
| Venezuela | - | - | 100.00 | 0.00 | 0.00 | 0.00 | 0.00 | 0.00 | 0.00 | 100.00 | 88.68 | 100.00 | 98.08 | 98.08 | 98.08 | 52.53 |
| **Average** | - | - | **3.78** | **24.01** | **36.04** | **45.23** | **37.33** | **31.90** | **54.38** | **75.27** | **54.46** | **50.66** | **63.00** | **67.11** | **67.64** | **46.98** |

| **Influenza B(Victoria) Positives** | | | | | | | | | | | | | | | | |
| --- | --- | --- | --- | --- | --- | --- | --- | --- | --- | --- | --- | --- | --- | --- | --- | --- |
| Argentina | - | - | 0.00 | 96.15 | 96.23 | 100.00 | 100.00 | 100.00 | 100.00 | 100.00 | 100.00 | 100.00 | 100.00 | 100.00 | 100.00 | 91.72 |
| Barbados | - | - | 0.00 | 0.00 | 0.00 | 0.00 | 0.00 | 0.00 | 0.00 | 0.00 | 0.00 | 11.54 | 0.00 | 0.00 | 13.46 | 1.92 |
| Belize | - | - | 0.00 | 0.00 | 0.00 | 0.00 | 0.00 | 0.00 | 0.00 | 0.00 | 0.00 | 0.00 | 98.08 | 84.62 | 100.00 | 21.75 |
| Bolivia | - | - | 0.00 | 0.00 | 0.00 | 0.00 | 0.00 | 0.00 | 100.00 | 100.00 | 90.57 | 73.08 | 100.00 | 100.00 | 100.00 | 51.05 |
| Brazil | - | - | 7.69 | 100.00 | 100.00 | 100.00 | 100.00 | 100.00 | 100.00 | 100.00 | 100.00 | 78.85 | 46.15 | 90.38 | 90.38 | 85.65 |
| Canada | - | - | 0.00 | 0.00 | 1.89 | 84.62 | 98.08 | 98.08 | 100.00 | 100.00 | 100.00 | 51.92 | 0.00 | 0.00 | 0.00 | 48.81 |
| Chile | - | - | 0.00 | 21.15 | 33.96 | 100.00 | 100.00 | 100.00 | 100.00 | 100.00 | 50.94 | 92.31 | 100.00 | 100.00 | 100.00 | 76.80 |
| Colombia | - | - | 0.00 | 0.00 | 98.11 | 67.31 | 25.00 | 0.00 | 53.85 | 100.00 | 96.23 | 0.00 | 0.00 | 21.15 | 0.00 | 35.51 |
| Costa Rica | - | - | 0.00 | 88.46 | 30.19 | 23.08 | 42.31 | 19.23 | 46.15 | 100.00 | 0.00 | 0.00 | 0.00 | 0.00 | 100.00 | 34.57 |
| Cuba | - | - | 0.00 | 0.00 | 98.11 | 30.77 | 0.00 | 0.00 | 59.62 | 100.00 | 98.11 | 0.00 | 98.08 | 100.00 | 98.08 | 52.52 |
| Dominica | - | - | 0.00 | 0.00 | 0.00 | 0.00 | 0.00 | 0.00 | 0.00 | 0.00 | 0.00 | 0.00 | 0.00 | 13.46 | 0.00 | 1.04 |
| Dominican Republic | - | - | 0.00 | 0.00 | 0.00 | 0.00 | 0.00 | 0.00 | 50.00 | 96.15 | 0.00 | 100.00 | 28.85 | 13.46 | 5.77 | 22.63 |
| Ecuador | - | - | 0.00 | 0.00 | 3.77 | 0.00 | 0.00 | 0.00 | 50.00 | 100.00 | 0.00 | 21.15 | 0.00 | 100.00 | 0.00 | 21.15 |
| El Salvador | - | - | 0.00 | 50.00 | 84.91 | 86.54 | 96.15 | 76.92 | 80.77 | 100.00 | 100.00 | 100.00 | 100.00 | 96.15 | 100.00 | 82.42 |
| Guatemala | - | - | 0.00 | 0.00 | 33.96 | 92.31 | 15.38 | 7.69 | 100.00 | 94.23 | 98.11 | 100.00 | 92.31 | 26.92 | 46.15 | 54.39 |
| Haiti | - | - | 0.00 | 0.00 | 0.00 | 0.00 | 0.00 | 0.00 | 0.00 | 0.00 | 24.53 | 26.92 | 100.00 | 100.00 | 78.85 | 25.41 |
| Honduras | - | - | 0.00 | 32.69 | 98.11 | 100.00 | 100.00 | 28.85 | 88.46 | 92.31 | 0.00 | 100.00 | 98.08 | 100.00 | 98.08 | 72.04 |
| Jamaica | - | - | 0.00 | 100.00 | 98.11 | 100.00 | 69.23 | 1.92 | 48.08 | 100.00 | 100.00 | 61.54 | 100.00 | 98.08 | 100.00 | 75.15 |
| Mexico | - | - | 0.00 | 17.31 | 100.00 | 100.00 | 100.00 | 100.00 | 100.00 | 100.00 | 0.00 | 0.00 | 100.00 | 100.00 | 100.00 | 70.56 |
| Nicaragua | - | - | 0.00 | 0.00 | 0.00 | 0.00 | 0.00 | 0.00 | 48.08 | 100.00 | 1.89 | 0.00 | 0.00 | 0.00 | 0.00 | 11.54 |
| Panama | - | - | 0.00 | 98.08 | 20.75 | 100.00 | 100.00 | 98.08 | 100.00 | 100.00 | 100.00 | 100.00 | 100.00 | 100.00 | 100.00 | 85.92 |
| Paraguay | - | - | 0.00 | 96.15 | 100.00 | 26.92 | 0.00 | 0.00 | 51.92 | 100.00 | 98.11 | 100.00 | 100.00 | 100.00 | 100.00 | 67.16 |
| Peru | - | - | 0.00 | 0.00 | 0.00 | 100.00 | 36.54 | 94.23 | 42.31 | 100.00 | 100.00 | 98.08 | 100.00 | 82.69 | 100.00 | 65.68 |
| St. Lucia | - | - | 0.00 | 0.00 | 0.00 | 0.00 | 0.00 | 0.00 | 0.00 | 0.00 | 0.00 | 0.00 | 0.00 | 0.00 | 28.85 | 2.22 |
| St. Vincent & the Grenadines | - | - | 0.00 | 0.00 | 0.00 | 0.00 | 0.00 | 0.00 | 0.00 | 0.00 | 0.00 | 0.00 | 0.00 | 0.00 | 0.00 | 0.00 |
| Suriname | - | - | 0.00 | 0.00 | 0.00 | 0.00 | 0.00 | 0.00 | 0.00 | 0.00 | 98.11 | 100.00 | 55.77 | 98.08 | 96.15 | 34.47 |
| United States | - | - | 0.00 | 0.00 | 33.96 | 100.00 | 100.00 | 100.00 | 100.00 | 100.00 | 100.00 | 100.00 | 100.00 | 100.00 | 100.00 | 79.54 |
| Uruguay | - | - | 0.00 | 17.31 | 15.09 | 0.00 | 0.00 | 0.00 | 57.69 | 100.00 | 0.00 | 5.77 | 96.15 | 90.38 | 98.08 | 36.96 |
| Venezuela | - | - | 100.00 | 0.00 | 0.00 | 0.00 | 0.00 | 0.00 | 0.00 | 100.00 | 88.68 | 100.00 | 98.08 | 98.08 | 98.08 | 52.53 |
| **Average** | - | - | **3.71** | **24.73** | **36.11** | **45.23** | **37.33** | **31.90** | **54.38** | **75.27** | **53.29** | **52.45** | **62.47** | **65.98** | **67.31** | **46.93** |

| **Influenza B(Undetermined) Positives** | | | | | | | | | | | | | | | | |
| --- | --- | --- | --- | --- | --- | --- | --- | --- | --- | --- | --- | --- | --- | --- | --- | --- |
| Argentina | 98.08 | 100.00 | 94.23 | 96.15 | 96.23 | 100.00 | 100.00 | 100.00 | 100.00 | 100.00 | 100.00 | 100.00 | 100.00 | 100.00 | 100.00 | 98.98 |
| Barbados | 0.00 | 0.00 | 0.00 | 0.00 | 0.00 | 0.00 | 0.00 | 0.00 | 0.00 | 0.00 | 92.45 | 84.62 | 80.77 | 80.77 | 75.00 | 27.57 |
| Belize | 0.00 | 0.00 | 0.00 | 0.00 | 0.00 | 0.00 | 0.00 | 0.00 | 0.00 | 0.00 | 5.66 | 0.00 | 98.08 | 88.46 | 100.00 | 19.48 |
| Bolivia | 0.00 | 0.00 | 0.00 | 0.00 | 0.00 | 100.00 | 100.00 | 100.00 | 100.00 | 100.00 | 98.11 | 100.00 | 100.00 | 100.00 | 100.00 | 66.54 |
| Brazil | 100.00 | 100.00 | 100.00 | 100.00 | 100.00 | 100.00 | 100.00 | 100.00 | 100.00 | 100.00 | 100.00 | 100.00 | 100.00 | 100.00 | 98.08 | 99.87 |
| Canada | 0.00 | 34.62 | 96.15 | 100.00 | 86.79 | 100.00 | 98.08 | 98.08 | 100.00 | 100.00 | 100.00 | 100.00 | 100.00 | 100.00 | 100.00 | 87.58 |
| Chile | 100.00 | 100.00 | 98.08 | 94.23 | 96.23 | 100.00 | 100.00 | 100.00 | 100.00 | 100.00 | 54.72 | 59.62 | 100.00 | 100.00 | 100.00 | 93.52 |
| Colombia | 100.00 | 100.00 | 100.00 | 80.77 | 98.11 | 100.00 | 100.00 | 100.00 | 96.15 | 100.00 | 96.23 | 100.00 | 100.00 | 28.85 | 100.00 | 93.34 |
| Costa Rica | 5.77 | 21.15 | 40.38 | 100.00 | 41.51 | 100.00 | 100.00 | 100.00 | 100.00 | 100.00 | 98.11 | 100.00 | 100.00 | 98.08 | 100.00 | 80.33 |
| Cuba | 0.00 | 0.00 | 0.00 | 0.00 | 98.11 | 100.00 | 100.00 | 98.08 | 100.00 | 100.00 | 98.11 | 100.00 | 98.08 | 100.00 | 98.08 | 72.70 |
| Dominica | 0.00 | 0.00 | 0.00 | 0.00 | 0.00 | 0.00 | 0.00 | 0.00 | 0.00 | 0.00 | 49.06 | 0.00 | 42.31 | 44.23 | 63.46 | 13.27 |
| Dominican Republic | 100.00 | 100.00 | 100.00 | 100.00 | 16.98 | 100.00 | 100.00 | 100.00 | 100.00 | 96.15 | 98.11 | 100.00 | 78.85 | 69.23 | 80.77 | 89.34 |
| Ecuador | 0.00 | 0.00 | 3.85 | 0.00 | 64.15 | 88.46 | 100.00 | 100.00 | 100.00 | 100.00 | 92.45 | 96.15 | 100.00 | 100.00 | 100.00 | 69.67 |
| El Salvador | 0.00 | 82.69 | 0.00 | 53.85 | 84.91 | 96.15 | 100.00 | 100.00 | 100.00 | 100.00 | 100.00 | 100.00 | 100.00 | 96.15 | 100.00 | 80.92 |
| Guatemala | 0.00 | 0.00 | 0.00 | 0.00 | 33.96 | 98.08 | 100.00 | 96.15 | 100.00 | 94.23 | 98.11 | 100.00 | 92.31 | 86.54 | 57.69 | 63.81 |
| Haiti | 0.00 | 0.00 | 0.00 | 0.00 | 0.00 | 0.00 | 0.00 | 0.00 | 0.00 | 0.00 | 24.53 | 26.92 | 100.00 | 100.00 | 92.31 | 22.92 |
| Honduras | 0.00 | 0.00 | 100.00 | 98.08 | 98.11 | 100.00 | 100.00 | 96.15 | 98.08 | 92.31 | 98.11 | 100.00 | 98.08 | 100.00 | 98.08 | 85.13 |
| Jamaica | 0.00 | 0.00 | 0.00 | 100.00 | 98.11 | 100.00 | 98.08 | 100.00 | 100.00 | 100.00 | 100.00 | 92.31 | 100.00 | 98.08 | 100.00 | 79.10 |
| Mexico | 100.00 | 100.00 | 100.00 | 100.00 | 100.00 | 100.00 | 100.00 | 100.00 | 100.00 | 100.00 | 98.11 | 100.00 | 100.00 | 100.00 | 100.00 | 99.87 |
| Nicaragua | 0.00 | 0.00 | 0.00 | 0.00 | 0.00 | 100.00 | 100.00 | 100.00 | 100.00 | 100.00 | 100.00 | 100.00 | 100.00 | 100.00 | 100.00 | 66.67 |
| Panama | 0.00 | 0.00 | 100.00 | 100.00 | 24.53 | 100.00 | 100.00 | 100.00 | 100.00 | 100.00 | 100.00 | 100.00 | 100.00 | 100.00 | 100.00 | 81.64 |
| Paraguay | 100.00 | 30.77 | 0.00 | 98.08 | 100.00 | 100.00 | 100.00 | 100.00 | 100.00 | 100.00 | 98.11 | 100.00 | 100.00 | 100.00 | 100.00 | 88.46 |
| Peru | 100.00 | 100.00 | 100.00 | 98.08 | 39.62 | 100.00 | 100.00 | 100.00 | 100.00 | 100.00 | 100.00 | 98.08 | 100.00 | 82.69 | 100.00 | 94.56 |
| St. Lucia | 0.00 | 0.00 | 0.00 | 0.00 | 0.00 | 0.00 | 0.00 | 0.00 | 0.00 | 0.00 | 26.42 | 0.00 | 44.23 | 71.15 | 63.46 | 13.68 |
| St. Vincent & the Grenadines | 0.00 | 0.00 | 0.00 | 0.00 | 0.00 | 0.00 | 0.00 | 0.00 | 0.00 | 0.00 | 3.77 | 0.00 | 3.85 | 21.15 | 65.38 | 6.28 |
| Suriname | 0.00 | 0.00 | 0.00 | 0.00 | 0.00 | 0.00 | 0.00 | 0.00 | 0.00 | 0.00 | 98.11 | 100.00 | 98.08 | 98.08 | 96.15 | 32.69 |
| United States | 100.00 | 63.46 | 100.00 | 100.00 | 100.00 | 100.00 | 100.00 | 100.00 | 100.00 | 100.00 | 100.00 | 100.00 | 100.00 | 100.00 | 100.00 | 97.56 |
| Uruguay | 51.92 | 46.15 | 63.46 | 59.62 | 47.17 | 50.00 | 71.15 | 75.00 | 100.00 | 100.00 | 98.11 | 92.31 | 96.15 | 90.38 | 98.08 | 75.97 |
| Venezuela | 15.38 | 67.31 | 100.00 | 0.00 | 0.00 | 0.00 | 100.00 | 0.00 | 0.00 | 100.00 | 88.68 | 100.00 | 98.08 | 98.08 | 98.08 | 57.71 |
| **Average** | **33.49** | **36.07** | **44.69** | **50.99** | **49.12** | **70.09** | **74.73** | **71.15** | **72.21** | **75.27** | **83.28** | **81.03** | **90.65** | **88.00** | **92.57** | **67.56** |

| **Negatives** | | | | | | | | | | | | | | | | |
| --- | --- | --- | --- | --- | --- | --- | --- | --- | --- | --- | --- | --- | --- | --- | --- | --- |
| Argentina | 90.38 | 84.62 | 86.54 | 88.46 | 73.58 | 100.00 | 100.00 | 100.00 | 100.00 | 100.00 | 100.00 | 100.00 | 100.00 | 100.00 | 100.00 | 94.91 |
| Barbados | 0.00 | 0.00 | 0.00 | 0.00 | 0.00 | 0.00 | 0.00 | 0.00 | 0.00 | 0.00 | 92.45 | 88.46 | 80.77 | 84.62 | 78.85 | 28.34 |
| Belize | 0.00 | 0.00 | 0.00 | 0.00 | 0.00 | 0.00 | 0.00 | 0.00 | 0.00 | 0.00 | 5.66 | 0.00 | 98.08 | 88.46 | 100.00 | 19.48 |
| Bolivia | 0.00 | 0.00 | 0.00 | 0.00 | 0.00 | 100.00 | 100.00 | 100.00 | 100.00 | 100.00 | 98.11 | 100.00 | 100.00 | 100.00 | 100.00 | 66.54 |
| Brazil | 100.00 | 98.08 | 100.00 | 96.15 | 90.57 | 100.00 | 100.00 | 100.00 | 100.00 | 100.00 | 100.00 | 78.85 | 100.00 | 100.00 | 98.08 | 97.45 |
| Canada | 0.00 | 0.00 | 0.00 | 0.00 | 0.00 | 0.00 | 0.00 | 0.00 | 0.00 | 0.00 | 0.00 | 0.00 | 0.00 | 0.00 | 0.00 | 0.00 |
| Chile | 100.00 | 100.00 | 98.08 | 100.00 | 96.23 | 100.00 | 100.00 | 100.00 | 100.00 | 100.00 | 100.00 | 100.00 | 100.00 | 100.00 | 100.00 | 99.62 |
| Colombia | 100.00 | 100.00 | 100.00 | 78.85 | 98.11 | 100.00 | 100.00 | 100.00 | 96.15 | 100.00 | 96.23 | 100.00 | 100.00 | 100.00 | 100.00 | 97.96 |
| Costa Rica | 0.00 | 0.00 | 0.00 | 71.15 | 41.51 | 98.08 | 100.00 | 100.00 | 98.08 | 98.08 | 98.11 | 100.00 | 98.08 | 98.08 | 100.00 | 73.41 |
| Cuba | 0.00 | 0.00 | 0.00 | 0.00 | 96.23 | 100.00 | 100.00 | 98.08 | 100.00 | 100.00 | 98.11 | 100.00 | 98.08 | 100.00 | 98.08 | 72.57 |
| Dominica | 0.00 | 0.00 | 0.00 | 0.00 | 0.00 | 0.00 | 0.00 | 0.00 | 0.00 | 0.00 | 52.83 | 0.00 | 42.31 | 53.85 | 34.62 | 12.24 |
| Dominican Republic | 57.69 | 75.00 | 51.92 | 92.31 | 16.98 | 100.00 | 100.00 | 100.00 | 100.00 | 94.23 | 96.23 | 100.00 | 98.08 | 100.00 | 100.00 | 85.50 |
| Ecuador | 0.00 | 0.00 | 3.85 | 0.00 | 32.08 | 88.46 | 100.00 | 100.00 | 100.00 | 100.00 | 98.11 | 100.00 | 100.00 | 100.00 | 100.00 | 68.17 |
| El Salvador | 0.00 | 63.46 | 0.00 | 50.00 | 83.02 | 96.15 | 100.00 | 100.00 | 100.00 | 98.08 | 100.00 | 100.00 | 100.00 | 96.15 | 100.00 | 79.12 |
| Guatemala | 0.00 | 0.00 | 0.00 | 0.00 | 30.19 | 98.08 | 98.08 | 96.15 | 100.00 | 94.23 | 96.23 | 100.00 | 92.31 | 100.00 | 100.00 | 67.02 |
| Haiti | 0.00 | 0.00 | 0.00 | 0.00 | 0.00 | 0.00 | 0.00 | 0.00 | 0.00 | 0.00 | 24.53 | 26.92 | 100.00 | 100.00 | 78.85 | 22.02 |
| Honduras | 0.00 | 0.00 | 67.31 | 88.46 | 96.23 | 100.00 | 100.00 | 96.15 | 98.08 | 92.31 | 98.11 | 100.00 | 98.08 | 100.00 | 98.08 | 82.19 |
| Jamaica | 0.00 | 0.00 | 0.00 | 0.00 | 98.11 | 100.00 | 94.23 | 100.00 | 94.23 | 94.23 | 94.34 | 96.15 | 100.00 | 98.08 | 100.00 | 71.29 |
| Mexico | 100.00 | 100.00 | 100.00 | 100.00 | 100.00 | 100.00 | 100.00 | 100.00 | 100.00 | 100.00 | 98.11 | 100.00 | 100.00 | 100.00 | 100.00 | 99.87 |
| Nicaragua | 0.00 | 0.00 | 0.00 | 0.00 | 0.00 | 100.00 | 100.00 | 100.00 | 100.00 | 100.00 | 100.00 | 100.00 | 100.00 | 100.00 | 100.00 | 66.67 |
| Panama | 0.00 | 0.00 | 96.15 | 98.08 | 24.53 | 100.00 | 100.00 | 100.00 | 100.00 | 100.00 | 100.00 | 100.00 | 100.00 | 100.00 | 100.00 | 81.25 |
| Paraguay | 98.08 | 30.77 | 0.00 | 90.38 | 98.11 | 100.00 | 100.00 | 100.00 | 100.00 | 100.00 | 96.23 | 100.00 | 100.00 | 100.00 | 100.00 | 87.57 |
| Peru | 100.00 | 100.00 | 100.00 | 98.08 | 37.74 | 100.00 | 100.00 | 100.00 | 100.00 | 100.00 | 100.00 | 98.08 | 100.00 | 82.69 | 100.00 | 94.44 |
| St. Lucia | 0.00 | 0.00 | 0.00 | 0.00 | 0.00 | 0.00 | 0.00 | 0.00 | 0.00 | 0.00 | 26.42 | 0.00 | 44.23 | 73.08 | 17.31 | 10.74 |
| St. Vincent & the Grenadines | 0.00 | 0.00 | 0.00 | 0.00 | 0.00 | 0.00 | 0.00 | 0.00 | 0.00 | 0.00 | 3.77 | 0.00 | 3.85 | 15.38 | 36.54 | 3.97 |
| Suriname | 0.00 | 0.00 | 0.00 | 0.00 | 0.00 | 0.00 | 0.00 | 0.00 | 0.00 | 0.00 | 88.68 | 100.00 | 98.08 | 98.08 | 96.15 | 32.07 |
| United States | 0.00 | 0.00 | 0.00 | 0.00 | 0.00 | 0.00 | 0.00 | 0.00 | 0.00 | 0.00 | 24.53 | 100.00 | 100.00 | 100.00 | 100.00 | 28.30 |
| Uruguay | 50.00 | 36.54 | 59.62 | 51.92 | 47.17 | 50.00 | 69.23 | 75.00 | 86.54 | 96.15 | 77.36 | 98.08 | 96.15 | 90.38 | 96.15 | 72.02 |
| Venezuela | 3.85 | 42.31 | 98.08 | 0.00 | 0.00 | 0.00 | 100.00 | 0.00 | 0.00 | 98.08 | 88.68 | 98.08 | 98.08 | 96.15 | 76.92 | 53.35 |
| **Average** | **27.59** | **28.65** | **33.16** | **38.06** | **40.01** | **63.13** | **67.64** | **64.32** | **64.59** | **67.77** | **77.68** | **78.78** | **87.80** | **88.79** | **86.54** | **60.97** |

| **Specimens** | | | | | | | | | | | | | | | | |
| --- | --- | --- | --- | --- | --- | --- | --- | --- | --- | --- | --- | --- | --- | --- | --- | --- |
| Argentina | - | - | - | 0.00 | 73.58 | 63.46 | 75.00 | 100.00 | 100.00 | 61.54 | 66.04 | 53.85 | 0.00 | 0.00 | 0.00 | 49.46 |
| Barbados | - | - | - | 0.00 | 0.00 | 0.00 | 0.00 | 0.00 | 0.00 | 0.00 | 0.00 | 0.00 | 0.00 | 0.00 | 0.00 | 0.00 |
| Belize | - | - | - | 0.00 | 0.00 | 0.00 | 0.00 | 0.00 | 0.00 | 0.00 | 0.00 | 0.00 | 0.00 | 0.00 | 0.00 | 0.00 |
| Bolivia | - | - | - | 0.00 | 0.00 | 0.00 | 0.00 | 0.00 | 0.00 | 0.00 | 0.00 | 0.00 | 0.00 | 0.00 | 0.00 | 0.00 |
| Brazil | - | - | - | 0.00 | 67.92 | 100.00 | 100.00 | 100.00 | 100.00 | 100.00 | 100.00 | 100.00 | 100.00 | 7.69 | 0.00 | 72.97 |
| Canada | - | - | - | 0.00 | 0.00 | 82.69 | 98.08 | 98.08 | 100.00 | 100.00 | 100.00 | 100.00 | 100.00 | 100.00 | 100.00 | 81.57 |
| Chile | - | - | - | 0.00 | 73.58 | 100.00 | 100.00 | 100.00 | 55.77 | 0.00 | 0.00 | 0.00 | 0.00 | 0.00 | 0.00 | 35.78 |
| Colombia | - | - | - | 0.00 | 0.00 | 0.00 | 0.00 | 0.00 | 0.00 | 0.00 | 0.00 | 0.00 | 0.00 | 0.00 | 0.00 | 0.00 |
| Costa Rica | - | - | - | 0.00 | 0.00 | 23.08 | 42.31 | 19.23 | 0.00 | 0.00 | 0.00 | 0.00 | 0.00 | 0.00 | 0.00 | 7.05 |
| Cuba | - | - | - | 0.00 | 0.00 | 30.77 | 0.00 | 0.00 | 0.00 | 0.00 | 0.00 | 0.00 | 0.00 | 0.00 | 0.00 | 2.56 |
| Dominica | - | - | - | 0.00 | 0.00 | 0.00 | 0.00 | 0.00 | 0.00 | 0.00 | 0.00 | 0.00 | 0.00 | 0.00 | 0.00 | 0.00 |
| Dominican Republic | - | - | - | 0.00 | 0.00 | 0.00 | 0.00 | 0.00 | 0.00 | 0.00 | 0.00 | 0.00 | 0.00 | 0.00 | 0.00 | 0.00 |
| Ecuador | - | - | - | 0.00 | 32.08 | 0.00 | 0.00 | 0.00 | 0.00 | 0.00 | 0.00 | 0.00 | 0.00 | 0.00 | 0.00 | 2.67 |
| El Salvador | - | - | - | 0.00 | 84.91 | 86.54 | 96.15 | 76.92 | 65.38 | 86.54 | 49.06 | 0.00 | 0.00 | 0.00 | 0.00 | 45.46 |
| Guatemala | - | - | - | 0.00 | 33.96 | 92.31 | 3.85 | 7.69 | 65.38 | 0.00 | 0.00 | 0.00 | 0.00 | 0.00 | 0.00 | 16.93 |
| Haiti | - | - | - | 0.00 | 0.00 | 0.00 | 0.00 | 0.00 | 0.00 | 0.00 | 0.00 | 0.00 | 0.00 | 0.00 | 0.00 | 0.00 |
| Honduras | - | - | - | 0.00 | 98.11 | 100.00 | 100.00 | 28.85 | 0.00 | 0.00 | 0.00 | 0.00 | 0.00 | 0.00 | 0.00 | 27.25 |
| Jamaica | - | - | - | 0.00 | 98.11 | 100.00 | 55.77 | 0.00 | 0.00 | 0.00 | 0.00 | 0.00 | 0.00 | 0.00 | 0.00 | 21.16 |
| Mexico | - | - | - | 0.00 | 0.00 | 0.00 | 0.00 | 0.00 | 0.00 | 0.00 | 0.00 | 0.00 | 0.00 | 0.00 | 0.00 | 0.00 |
| Nicaragua | - | - | - | 0.00 | 0.00 | 0.00 | 0.00 | 0.00 | 0.00 | 0.00 | 0.00 | 0.00 | 0.00 | 0.00 | 0.00 | 0.00 |
| Panama | - | - | - | 0.00 | 0.00 | 100.00 | 100.00 | 98.08 | 88.46 | 13.46 | 0.00 | 0.00 | 0.00 | 0.00 | 0.00 | 33.33 |
| Paraguay | - | - | - | 7.69 | 100.00 | 26.92 | 0.00 | 0.00 | 0.00 | 0.00 | 0.00 | 0.00 | 0.00 | 0.00 | 0.00 | 11.22 |
| Peru | - | - | - | 0.00 | 0.00 | 100.00 | 34.62 | 94.23 | 0.00 | 0.00 | 0.00 | 0.00 | 0.00 | 0.00 | 0.00 | 19.07 |
| St. Lucia | - | - | - | 0.00 | 0.00 | 0.00 | 0.00 | 0.00 | 0.00 | 0.00 | 0.00 | 0.00 | 0.00 | 0.00 | 0.00 | 0.00 |
| St. Vincent & the Grenadines | - | - | - | 0.00 | 0.00 | 0.00 | 0.00 | 0.00 | 0.00 | 0.00 | 0.00 | 0.00 | 0.00 | 0.00 | 0.00 | 0.00 |
| Suriname | - | - | - | 0.00 | 0.00 | 0.00 | 0.00 | 0.00 | 0.00 | 0.00 | 0.00 | 0.00 | 0.00 | 0.00 | 0.00 | 0.00 |
| United States | - | - | - | 0.00 | 100.00 | 100.00 | 100.00 | 100.00 | 100.00 | 100.00 | 100.00 | 100.00 | 100.00 | 100.00 | 100.00 | 91.67 |
| Uruguay | - | - | - | 0.00 | 30.19 | 0.00 | 0.00 | 0.00 | 0.00 | 0.00 | 0.00 | 0.00 | 0.00 | 0.00 | 0.00 | 2.52 |
| Venezuela | - | - | - | 0.00 | 0.00 | 0.00 | 0.00 | 0.00 | 0.00 | 100.00 | 67.92 | 0.00 | 0.00 | 0.00 | 0.00 | 13.99 |
| **Average** | - | - | - | **0.27** | **27.33** | **38.13** | **31.23** | **28.38** | **23.28** | **19.36** | **16.66** | **12.20** | **10.34** | **7.16** | **6.90** | **18.44** |

**Supplementary Table S5.** Annual completeness for 14 influenza variables from 2005-2019 for Venezuela. For 5 variables (A(H1N1)pdm09, A(H5), B(Yamagata), B(Victoria), and Specimens) average completeness values were corrected as shown in Table 2.

| Variable | 2005 | 2006 | 2007 | 2008 | 2009 | 2010 | 2011 | 2012 | 2013 | 2014 | 2015 | 2016 | 2017 | 2018 | 2019 | Average |
| --- | --- | --- | --- | --- | --- | --- | --- | --- | --- | --- | --- | --- | --- | --- | --- | --- |
| **Venezuela** | | | | | | | | | | | | | | | | |
| Tests | 3.85 | 42.31 | 100.00 | 0.00 | 0.00 | 0.00 | 100.00 | 0.00 | 0.00 | 100.00 | 88.68 | 100.00 | 98.08 | 98.08 | 98.08 | 55.27 |
| Positives | 15.38 | 67.31 | 100.00 | 0.00 | 0.00 | 0.00 | 100.00 | 0.00 | 0.00 | 100.00 | 88.68 | 100.00 | 98.08 | 98.08 | 98.08 | 57.71 |
| Flu A | 15.38 | 67.31 | 100.00 | 0.00 | 0.00 | 0.00 | 100.00 | 0.00 | 0.00 | 100.00 | 88.68 | 100.00 | 98.08 | 98.08 | 98.08 | 57.71 |
| Flu B | 15.38 | 67.31 | 100.00 | 0.00 | 0.00 | 0.00 | 100.00 | 0.00 | 0.00 | 100.00 | 88.68 | 100.00 | 98.08 | 98.08 | 98.08 | 57.71 |
| A(H1) | 0.00 | 32.69 | 98.08 | 0.00 | 0.00 | 0.00 | 100.00 | 0.00 | 0.00 | 100.00 | 88.68 | 100.00 | 98.08 | 98.08 | 98.08 | 54.25 |
| A(H1N1)pdm09 | 0.00 | 0.00 | 0.00 | 0.00 | 0.00 | 0.00 | 100.00 | 0.00 | 0.00 | 100.00 | 88.68 | 100.00 | 98.08 | 98.08 | 98.08 | 56.91 |
| A(H5) | 0.00 | 0.00 | 0.00 | 0.00 | 0.00 | 0.00 | 0.00 | 0.00 | 0.00 | 100.00 | 67.92 | 0.00 | 0.00 | 0.00 | 0.00 | 18.66 |
| A(H3) | 15.38 | 13.46 | 100.00 | 0.00 | 0.00 | 0.00 | 100.00 | 0.00 | 0.00 | 100.00 | 88.68 | 100.00 | 98.08 | 98.08 | 98.08 | 54.12 |
| A(Unsubtyped) | 1.92 | 0.00 | 100.00 | 0.00 | 0.00 | 0.00 | 100.00 | 0.00 | 0.00 | 100.00 | 88.68 | 100.00 | 98.08 | 98.08 | 98.08 | 52.32 |
| B(Yamataga) | 0.00 | 0.00 | 100.00 | 0.00 | 0.00 | 0.00 | 0.00 | 0.00 | 0.00 | 100.00 | 88.68 | 100.00 | 98.08 | 98.08 | 98.08 | 52.53 |
| B(Victoria) | 0.00 | 0.00 | 100.00 | 0.00 | 0.00 | 0.00 | 0.00 | 0.00 | 0.00 | 100.00 | 88.68 | 100.00 | 98.08 | 98.08 | 98.08 | 52.53 |
| B(Undetermined) | 15.38 | 67.31 | 100.00 | 0.00 | 0.00 | 0.00 | 100.00 | 0.00 | 0.00 | 100.00 | 88.68 | 100.00 | 98.08 | 98.08 | 98.08 | 57.71 |
| Negatives | 3.85 | 42.31 | 98.08 | 0.00 | 0.00 | 0.00 | 100.00 | 0.00 | 0.00 | 98.08 | 88.68 | 98.08 | 98.08 | 96.15 | 76.92 | 53.35 |
| Specimens | 0.00 | 0.00 | 0.00 | 0.00 | 0.00 | 0.00 | 0.00 | 0.00 | 0.00 | 100.00 | 67.92 | 0.00 | 0.00 | 0.00 | 0.00 | 13.99 |
| **Average** | **6.18** | **28.57** | **78.30** | **0.00** | **0.00** | **0.00** | **71.43** | **0.00** | **0.00** | **99.86** | **85.71** | **85.58** | **84.07** | **83.93** | **82.55** | **49.63** |

**Supplementary Table S6.** The average completeness of 14 influenza variables and overall from 2005-2019 for 29 Pan American countries and all countries combined. For 5 variables (A(H1N1)pdm09, A(H5), B(Yamagata), B(Victoria), and Specimens) average completeness values were corrected as shown in Table 2.

| Country | Test* | Pos* | Flu A* | Flu B* | A(H1)* | A(H1N1)* | A(H3)* | A(H5)* | A(Uns.)* | B(Yam.)* | B(Vic.)* | B(Und.)* | Neg.* | Spec.* | Overall* |
| --- | --- | --- | --- | --- | --- | --- | --- | --- | --- | --- | --- | --- | --- | --- | --- |
| Argentina | 98.98 | 98.98 | 98.98 | 98.98 | 89.11 | 89.47 | 98.34 | 79.77 | 98.34 | 91.72 | 91.72 | 98.98 | 94.91 | 49.46 | 91.27 |
| Barbados | 28.47 | 28.47 | 28.47 | 28.47 | 0.00 | 87.71 | 2.69 | 0.00 | 0.25 | 0.59 | 1.92 | 27.57 | 28.34 | 0.00 | 18.78 |
| Belize | 19.48 | 19.48 | 19.48 | 19.48 | 18.85 | 4.30 | 18.85 | 0.00 | 18.85 | 21.75 | 21.75 | 19.48 | 19.48 | 0.00 | 15.80 |
| Bolivia | 66.54 | 66.54 | 66.54 | 66.54 | 64.24 | 23.56 | 64.37 | 0.00 | 57.58 | 51.05 | 51.05 | 66.54 | 66.54 | 0.00 | 50.79 |
| Brazil | 98.46 | 99.87 | 99.87 | 99.87 | 81.03 | 80.94 | 98.72 | 82.97 | 86.41 | 84.62 | 85.65 | 99.87 | 97.45 | 72.97 | 90.62 |
| Canada | 87.58 | 87.58 | 87.58 | 87.58 | 51.81 | 90.25 | 74.38 | 70.51 | 87.45 | 48.81 | 48.81 | 87.58 | 0.00 | 81.57 | 70.82 |
| Chile | 99.62 | 99.62 | 99.62 | 99.62 | 66.99 | 84.09 | 74.07 | 43.30 | 83.35 | 75.87 | 76.80 | 93.52 | 99.62 | 35.78 | 80.85 |
| Colombia | 98.08 | 98.08 | 98.08 | 98.08 | 59.37 | 90.87 | 79.37 | 21.16 | 77.70 | 47.35 | 35.51 | 93.34 | 97.96 | 0.00 | 71.07 |
| Costa Rica | 78.28 | 80.33 | 80.33 | 80.33 | 74.84 | 83.17 | 75.86 | 9.40 | 77.90 | 34.57 | 34.57 | 80.33 | 73.41 | 7.05 | 62.17 |
| Cuba | 72.70 | 72.70 | 72.70 | 72.70 | 72.70 | 90.87 | 72.70 | 14.32 | 72.70 | 52.52 | 52.52 | 72.70 | 72.57 | 2.56 | 61.92 |
| Dominica | 14.29 | 14.29 | 14.29 | 14.29 | 0.00 | 1.91 | 0.51 | 0.00 | 0.00 | 0.29 | 1.04 | 13.27 | 12.24 | 0.00 | 6.17 |
| Dominican Republic | 94.08 | 94.08 | 94.08 | 94.08 | 46.41 | 65.39 | 50.38 | 0.00 | 75.11 | 22.04 | 22.63 | 89.34 | 85.50 | 0.00 | 59.51 |
| Ecuador | 68.17 | 70.30 | 70.30 | 70.30 | 41.50 | 64.34 | 55.77 | 0.42 | 35.10 | 19.96 | 21.15 | 69.67 | 68.17 | 2.67 | 46.99 |
| El Salvador | 80.92 | 80.92 | 80.92 | 80.92 | 80.79 | 2.72 | 80.79 | 51.18 | 80.15 | 82.42 | 82.42 | 80.92 | 79.12 | 45.46 | 70.69 |
| Guatemala | 67.52 | 67.52 | 67.52 | 67.52 | 54.06 | 89.77 | 61.50 | 23.86 | 58.42 | 50.54 | 54.39 | 63.81 | 67.02 | 16.93 | 57.88 |
| Haiti | 22.92 | 22.92 | 22.92 | 22.92 | 20.22 | 27.52 | 22.02 | 0.00 | 20.22 | 25.41 | 25.41 | 22.92 | 22.02 | 0.00 | 19.82 |
| Honduras | 85.00 | 85.13 | 85.13 | 85.13 | 72.06 | 78.96 | 85.13 | 36.33 | 85.13 | 72.04 | 72.04 | 85.13 | 82.19 | 27.25 | 74.05 |
| Jamaica | 72.69 | 79.36 | 79.36 | 79.36 | 79.36 | 89.91 | 79.36 | 41.24 | 79.36 | 75.15 | 75.15 | 79.10 | 71.29 | 21.16 | 71.56 |
| Mexico | 99.87 | 99.87 | 99.87 | 99.87 | 99.75 | 91.67 | 99.75 | 70.63 | 99.49 | 70.56 | 70.56 | 99.87 | 99.87 | 0.00 | 85.83 |
| Nicaragua | 66.67 | 66.67 | 66.67 | 66.67 | 33.46 | 52.85 | 43.56 | 0.00 | 37.05 | 11.54 | 11.54 | 66.67 | 66.67 | 0.00 | 42.14 |
| Panama | 81.64 | 81.64 | 81.64 | 81.64 | 74.72 | 83.49 | 74.59 | 45.30 | 77.79 | 85.92 | 85.92 | 81.64 | 81.25 | 33.33 | 75.03 |
| Paraguay | 88.46 | 88.46 | 88.46 | 88.46 | 86.28 | 81.73 | 87.18 | 14.96 | 82.82 | 67.16 | 67.16 | 88.46 | 87.57 | 11.22 | 73.46 |
| Peru | 94.44 | 94.56 | 94.56 | 94.56 | 75.63 | 92.15 | 75.13 | 25.64 | 93.92 | 65.68 | 65.68 | 94.56 | 94.44 | 19.07 | 77.15 |
| St. Lucia | 13.81 | 13.81 | 13.81 | 13.81 | 1.67 | 99.20 | 2.05 | 0.00 | 1.67 | 2.07 | 2.22 | 13.68 | 10.74 | 0.00 | 13.47 |
| St. Vincent & the Grenadines | 6.28 | 6.28 | 6.28 | 6.28 | 0.00 | 69.18 | 0.64 | 0.00 | 0.00 | 0.00 | 0.00 | 6.28 | 3.97 | 0.00 | 7.51 |
| Suriname | 32.82 | 32.82 | 32.82 | 32.82 | 29.75 | 1.28 | 30.39 | 0.00 | 29.75 | 34.32 | 34.47 | 32.69 | 32.07 | 0.00 | 25.43 |
| United States | 97.56 | 97.56 | 97.56 | 97.56 | 97.56 | 89.47 | 97.56 | 67.71 | 97.56 | 79.54 | 79.54 | 97.56 | 28.30 | 91.67 | 86.91 |
| Uruguay | 76.48 | 76.48 | 76.48 | 76.48 | 66.26 | 37.18 | 66.13 | 0.00 | 66.23 | 36.52 | 36.96 | 75.97 | 72.02 | 2.52 | 54.69 |
| Venezuela | 55.27 | 57.71 | 57.71 | 57.71 | 54.25 | 56.91 | 54.12 | 18.66 | 52.32 | 52.53 | 52.53 | 57.71 | 53.35 | 13.99 | 49.63 |
| **Average** | **67.83** | **68.35** | **68.35** | **68.35** | **54.92** | **65.55** | **59.51** | **24.74** | **59.75** | **46.98** | **46.93** | **67.56** | **60.97** | **18.44** | **55.59** |

* Outcomes: total specimens processed (*Test*), total positives (*Pos*), influenza A positives (*Flu A*), influenza B positives (*Flu B*), total negatives (*Neg.*), and total specimens collected (*Spec.*). Subtypes of influenza positives: A(H1), A(H1N1)pdm09 *(A(H1N1))*, A(H3), A(H5), A(Unsubtyped) *(A(Uns.))*, B(Yamataga) *(B(Yam.))*, B(Victorian) *(B(Vic.))*, and B(Undetermined) *(B(Und.))*. *Overall* provides the average completeness across all influenza variables. *Average* provides the average completeness for each variable across all 29 countries.

**Supplementary Table S7.** The decadal values and change (2005-2015) in three economic indicators (GNI per capita (GNIPC), domestic health expenditure per capita (DHEPC), and out-of-pocket health expenditure as a percentage of total health expenditure (OOPHE%)) for 29 Pan American countries. *Average* refers to the average decadal change across all countries. *Difference* refers to the percentage difference between 2005 and 2015 values. *Ratio* refers to the quotient of the 2015 and 2005 values.

|  | **GNIPC*** | | | **DHEPC*** | | | **OOPHE%*** | | |
| --- | --- | --- | --- | --- | --- | --- | --- | --- | --- |
| **Country** | **2005** | **2015** | **Ratio** | **2005** | **2015** | **Ratio** | **2005** | **2015** | **Difference** |
| Argentina | 4260 | 12600 | 2.96 | 540.74 | 1411.63 | 2.11 | 32.99 | 13.55 | 0.48 |
| Barbados | 13520 | 15270 | 1.13 | 546.18 | 577.02 | 1.11 | 35.07 | 45.23 | 1.29 |
| Belize | 3670 | 4420 | 1.20 | 169.93 | 356.32 | 2.11 | 33.98 | 23.42 | 0.67 |
| Bolivia | 1020 | 2960 | 2.90 | 118.74 | 303.97 | 2.75 | 30.47 | 25.87 | 0.92 |
| Brazil | 4000 | 10160 | 2.54 | 388.99 | 593.11 | 1.52 | 34.98 | 44.68 | 1.25 |
| Canada | 34800 | 47590 | 1.37 | 2394.35 | 3401.46 | 1.45 | 15.37 | 14.47 | 0.95 |
| Chile | 6210 | 14140 | 2.28 | 445.50 | 1111.02 | 2.63 | 42.47 | 34.51 | 0.82 |
| Colombia | 2990 | 7330 | 2.45 | 329.68 | 528.29 | 1.60 | 17.89 | 19.58 | 1.13 |
| Costa Rica | 4620 | 10400 | 2.25 | 425.49 | 916.11 | 2.19 | 32.77 | 22.74 | 0.68 |
| Cuba | 3960 | 7230 | 1.83 | 905.76 | 2831.58 | 2.43 | 14.85 | 9.64 | 0.69 |
| Dominica | 5050 | 6670 | 1.32 | 234.57 | 378.32 | 1.59 | 40.45 | 31.17 | 0.72 |
| Dominican Republic | 3080 | 6580 | 2.14 | 115.67 | 352.28 | 3.70 | 53.88 | 42.85 | 0.83 |
| Ecuador | 2880 | 5970 | 2.07 | 128.97 | 485.45 | 3.74 | 63.01 | 43.87 | 0.64 |
| El Salvador | 2420 | 3440 | 1.42 | 226.47 | 368.76 | 1.71 | 43.04 | 27.85 | 0.63 |
| Guatemala | 2010 | 3620 | 1.80 | 109.74 | 149.89 | 1.57 | 62.79 | 57.82 | 0.85 |
| Haiti | 410 | 820 | 2.00 | 13.52 | 15.29 | 1.08 | 46.02 | 41.73 | 0.91 |
| Honduras | 1280 | 2060 | 1.61 | 96.12 | 137.40 | 1.91 | 47.94 | 49.15 | 0.94 |
| Jamaica | 3780 | 4710 | 1.25 | 173.99 | 308.83 | 1.86 | 29.85 | 18.87 | 0.75 |
| Mexico | 8050 | 10170 | 1.26 | 303.60 | 516.01 | 1.67 | 54.59 | 40.19 | 0.74 |
| Nicaragua | 1160 | 1910 | 1.65 | 87.86 | 238.60 | 3.38 | 44.22 | 35.60 | 0.73 |
| Panama | 4840 | 11740 | 2.43 | 410.62 | 971.07 | 2.80 | 32.22 | 30.22 | 0.85 |
| Paraguay | 1520 | 5620 | 3.70 | 106.22 | 385.43 | 3.73 | 50.62 | 36.49 | 0.75 |
| Peru | 2540 | 6340 | 2.50 | 166.71 | 411.72 | 2.62 | 34.54 | 28.95 | 0.82 |
| St. Lucia | 5480 | 7850 | 1.43 | 186.05 | 277.96 | 1.53 | 55.93 | 52.25 | 0.87 |
| St. Vincent & the Grenadines | 4960 | 6560 | 1.32 | 210.12 | 292.66 | 1.49 | 21.61 | 20.88 | 0.95 |
| Suriname | 3320 | 8690 | 2.62 | 341.55 | 635.25 | 1.84 | 18.13 | 20.24 | 1.20 |
| United States | 46190 | 56720 | 1.23 | 2927.84 | 7805.12 | 2.76 | 13.86 | 11.14 | 0.80 |
| Uruguay | 4720 | 15640 | 3.31 | 439.67 | 1301.27 | 3.19 | 25.94 | 16.92 | 0.67 |
| Venezuela** | 4980 | 13080 | 2.63 | 231.39 | 358.47 | 1.55 | 45.70 | 28.24 | 0.87 |
| **Average** | **9356.47** | **10699.66** | **2.02** | **440.55** | **945.53** | **2.19** | **37.08** | **30.62** | **0.84** |
| **Standard Deviation** | **11708.89** | **12016.58** | **0.67** | **635.40** | **1492.27** | **0.77** | **13.94** | **12.80** | **0.18** |

* GNIPC was reported in purchasing power parity (PPP) constant 2011 international US dollars (USD). DHEPC was reported in current international USD. OOPHE% is reported as the percentage of current government expenditure.

**** Percent difference and ratio estimates for GNIPC were calculated using 2005 and 2014 estimates. No data was available from 2015-2019.

**Appendix.** R codes for data scraping and merging.

**Scraper Code**

library(RSelenium)

#set up environment

driver <- RSelenium::rsDriver(browser = "chrome")

remote_driver <- driver[["client"]]

remote_driver$navigate('http://apps.who.int/flumart/Default?ReportNo=12')

#function to scrape data

scrape <- function(country, yearStart, yearTo) {

webElem1 <- remote_driver$findElement(using = "id", value = 'ddlFilterBy')

webElem2 <- remote_driver$findElement(using = "id", value = 'lstSearchBy')

webElem3 <- remote_driver$findElement(using = "id", value = 'ctl_list_YearFrom')

webElem4 <- remote_driver$findElement(using = "xpath", value = "/html/body/form/div[3]/fieldset/table/tbody/tr/td[1]/table/tbody/tr/td[14]/div/select/option[1]")

webElem5 <- remote_driver$findElement(using = "id", value = 'ctl_list_YearTo')

webElem6 <- remote_driver$findElement(using = "id", value = 'ctl_list_WeekTo')

webElem1$sendKeysToElement(list("1"))

webElem2$sendKeysToElement(list(country))

webElem3$sendKeysToElement(list(yearStart))

webElem4$clickElement()

webElem5$sendKeysToElement(list(yearTo))

webElem6$sendKeysToElement(list("53"))

webElem7 <- remote_driver$findElement(using = "css selector", value = "#ctl_ViewReport")

webElem7$clickElement()

Sys.sleep(40)

webElem8 <- remote_driver$findElement(using = "css selector", value = "#ctl_ReportViewer_ctl05_ctl04_ctl00_ButtonLink")

webElem8$clickElement()

Sys.sleep(3)

webElem9 <- remote_driver$findElement('xpath', "/html/body/form/div[3]/table/tbody/tr/td/span/div/table/tbody/tr[3]/td/div/div/div[4]/table/tbody/tr/td/div[2]/div[7]")

webElem9$clickElement()

Sys.sleep(20)}

**Merger Code:**

#to use this to clean and merge flunet files in csv

#click 'source' in rstudio to read in all the functions

#put all input files in an input folder, make an empty folder for output and an empty file for the merged file

#specify file and folder locations:

library(tidyverse)

library(lubridate)

abb <- function(txt){

read.delim(txt) -> key #lookup file for abbreviations

lapply(key[], as.character)-> key[]

key$WHO[key$WHO == ''] <- NA

return(key)}

#parameters <- function(inputFolder, outputFolder, finalFolder,WHO_abbreviations_file, startDate, endDate){

inputDir <- '/Users/Owner/Desktop/FluNet_World/input/'

outputDir <- '/Users/Owner/Desktop/FluNet_World/output/'

finalDir <- '/Users/Owner/Desktop/FluNet_World/final/'

key <- abb('/Users/Owner/Desktop/FluNet_World/computing/Key.txt')

start <- '12-31-2007'

end <- '12-29-2019'

setwd(inputDir)

#clean downloaded files

#clean()

#merge all cleaned files

#merge_all()

#Dependancies

#this is the list of column names

names<-c('Country','year','woy','start_date','end_date','spec', "test",

'ah1','ah1n1', 'ah3', 'ah5', 'aunsub', 'flua',

'byam', 'bvic', 'bunsub', 'flub',

'pos', 'neg', 'ILI')

#read in functions

#this functin reads in the csv, and skips the rows that are not data

df <- function(x){

try <- read.csv(x, skip = 5)

as.data.frame(try)-> try

return(try)

}

#this function checks the format of the date columns and standardizes

#the data is read in as object t and is hard coded that the dates are in column 3 and 4

#this function is a dependancy for the merge function

date_solution <- function(t){

lapply(t[3:4],as.character)-> t[3:4]

print(t[1,3:4])

if(str_detect(t[1,3], '-')) {print("-")} else{print("/")}

if(str_detect(t[1,3], '-')) {lapply(t[3],ymd) -> t[3]} else{lapply(t[3],mdy)-> t[3]}

if(str_detect(t[1,4], '-')) {lapply(t[4],ymd) -> t[4]} else{lapply(t[4],mdy)-> t[4]}

print(t[1,3:4])

print(unique(t[1]))

return(t)}

series <- function(){

s <- mdy(start)

e <- mdy(end)

n_days <- interval(s,e)/dweeks(1)

d <- s + dweeks(0:n_days)

d <- as.data.frame(d)

d$d + 6 -> d$end_date

colnames(d)[1]<- 'start_date'

week(d$start_date) -> d$woy

year(d$start_date) -> d$year

as.numeric(as.character(d$woy)) -> d$woy

as.numeric(as.character(d$year)) -> d$year

return(d)

}

clean <- function(){

list.files(inputDir, pattern = '.csv') -> x

series()-> d

for (i in x) {

df(i) -> r

r %>% mutate_all(na_if,"") -> r

r[,c(1,4:22)] -> r #this may need to be changed for new columns

colnames(r) <- names

as.character(r$Country) -> r$Country

key %>% filter(WHO == unique(r$Country)) %>% dplyr::select(X3.dig) -> try

try$X3.dig -> abbr

trimws(abbr, which = 'both') -> abbr

colnames(r)[6:20] <- paste(abbr, colnames(r)[6:20], sep = "_")#this may also need to be changed

NULL -> r$Country

as.numeric(as.character(r$woy)) -> r$woy

as.numeric(as.character(r$year)) -> r$year

merge(date_solution(r), d, by = c('year', 'woy', 'start_date', 'end_date'), all.y = T) -> r #this makes sure the time series is complete and checks date formatting

write.csv(r, paste0(outputDir,abbr,".csv", sep = ''), row.names = F)

print(cbind(i, nrow(r)))}

}

merge_all <- function(){

setwd(outputDir)

f <-list.files(pattern = 'csv')

rm(d)

d <- series()

for (i in f) {

print(i)

read.csv(i)-> i

as.numeric(as.character(i$woy)) -> i$woy

as.numeric(as.character(i$year)) -> i$year

if(exists('d')) {date_solution(i) -> new} else {date_solution(i) -> d}

merge(new, d, by = c('year', 'woy', 'start_date', 'end_date'), all = T) -> d

}

d[order(d$start_date),] -> A

A[is.na(A)] <- "."

write.csv(A, paste0(finalDir, Sys.Date(),'_countries.csv'), row.names = F)

return(A)}
